# Detection and Identification of Food-Borne Yeasts: An Overview of the Relevant Methods and Their Evolution

**DOI:** 10.3390/microorganisms13050981

**Published:** 2025-04-24

**Authors:** Mónika Kovács, Andrea Pomázi, Andrea Taczman-Brückner, Gabriella Kiskó, Viktória Dobó, Tamás Kocsis, Csilla Mohácsi-Farkas, Ágnes Belák

**Affiliations:** Department of Food Microbiology, Hygiene and Safety, Institute of Food Science and Technology, Hungarian University of Agriculture and Life Sciences, Somlói út 14-16, H-1118 Budapest, Hungary; kovacs.monika@uni-mate.hu (M.K.); pomazi.andrea@uni-mate.hu (A.P.); bruckner.andrea@uni-mate.hu (A.T.-B.); kisko.gabriella@uni-mate.hu (G.K.); dobo.viktoria@uni-mate.hu (V.D.); kocsis.tamas.jozsef@uni-mate.hu (T.K.); mohacsine.farkas.csilla@uni-mate.hu (C.M.-F.)

**Keywords:** yeasts, qualitative analysis, culture-based techniques, rapid detection, molecular methods

## Abstract

The presence of yeasts in food is not unexpected, as they are part of the microbiota of raw materials, employed as starter cultures in numerous fermentation processes, and also play a role in spontaneous fermentation. Nevertheless, they have the potential to induce spoilage, which can lead to significant quality issues, and certain yeasts have the ability to cause infections in humans and animals, posing a food safety risk. The detection of yeasts in food, determination of their cell number, as well as identification and typing, are therefore often tasks during the examination of certain food categories. The methods employed to achieve these objectives are diverse, encompassing both conventional culture-based techniques and more recent, genome-based studies. The objective of this study is to provide a summary article that presents the methods suitable for testing food-derived yeasts. The article will highlight the advantages, disadvantages, and potential difficulties of their applicability. Moreover, a comprehensive review of nucleic acid-based, culture-dependent and culture-independent molecular yeast identification techniques was conducted, encompassing scientific articles from the past five years (2020–2024). The search was based on the Science Direct database using the keywords “yeast and molecular identification and food”.

## 1. Introduction

Fungi have an important and multifaceted role in the production and processing of foodstuffs, exerting an influence on various stages from cultivation to consumption. Yeasts and moulds are integral to fermentation processes, yielding a wide array of fermented foods and beverages with unique flavours and textures. Furthermore, fungi are indispensable in the creation of enzymes and bioactive compounds that are utilised in food processing, enhancing the nutritional value, shelf life, and safety of food products. However, certain fungal species have the capacity to act as spoilage agents and mycotoxin producers, necessitating rigorous quality control measures [1].

Yeasts are prevalent in natural environments, where they play a significant role in the fermentation processes. They are common components of numerous traditional fermented foods, either as a solitary organism or as part of a stable association of mixed populations. Yeasts exert a substantial influence on the quality parameters of fermented food products, encompassing characteristics such as texture, taste, nutritional value, odour, and functional properties [2]. In the context of yeast genera reported from fermented foods and beverages, *Debaryomyces* has the highest documented prevalence, followed by *Candida*, *Saccharomyces*, *Pichia*, *Kluyveromyces*, *Wickerhamomyces*, *Toruplasora*, *Yarrowia,* and *Metschnikowia* [3].

*Saccharomyces cerevisiae* is the most frequently used yeast in the fermentation process of food products, and it has been shown to possess a range of technological properties [4]. *S. cerevisiae*’s usefulness is attributable to its unique biological characteristics, including its fermentation capacity, accompanied by the production of alcohol and CO_2_, and its resilience to adverse conditions of osmolarity and low pH [5]. In the field of industrial fermentation, this yeast is widely regarded as an essential organism, playing a central role in the production of diverse fermented products on a global scale. These include beverages such as beer, cider, wine, sake, distilled spirits, bakery items, cheese, sausages, and other fermented foods [5,6].

However, over the past two decades, both the research and industrial sectors have initiated a re-evaluation of the potential positive contributions of non-*Saccharomyces* yeasts (NSYs). These organisms have been identified as having a pro-technological use in traditional fermentations, where they have been shown to impart distinctive characteristics to the product. In addition to this, NSYs have been identified as having a role in a range of other applications, including biomedical or fundamental biological research, environmental biotechnology, heterologous protein production, biocontrol, and the food and feed sectors [7]. The focus on NSYs can be attributed to the presence of enzymes and secondary metabolites that are not typically produced by conventional *S. cerevisiae* yeast strains. Furthermore, the metabolic diversity exhibited by NSYs is now considered essential in order to respond to consumer demands for novel sensory properties and health benefits [6,7].

In addition to the fact that yeasts are regarded by the food industry as beneficial organisms, they are also used as biosensors due to various advantages, such as possession of specific receptors, stability, and high robustness [8]. The zymase activity of the baker’s yeast *S. cerevisiae* is sensitive to environmental parameters; consequently, it may be employed as a microbiological sensor. In comparison with the bacterial bioluminescence approach, this method is not toxic, does not require the use of genetically modified microorganisms, and enables low-cost, rapid analysis [9]. *S. cerevisiae*-based biosensors have been explored in various applications within the agri-food sector due to their robustness, genetic modifiability, and ability to detect contaminants, toxins, and environmental changes. Among others, *S. cerevisiae* and other yeasts can be used for the degradation of mycotoxins in food and feed [10], pesticide residue monitoring during bread fermentation [11], heavy metal detection in soil and water [12], antibiotic residue screening in milk and meat [13], monitoring fermentation processes in food and beverage production [14], and detection of hormone contaminants in food and water [15].

In recent years, there has been an increasing focus on the potential health benefits of yeasts as probiotics. Researchers are actively exploring new yeast strains with probiotic properties (e.g., *Debaryomyces hansenii*, *Kluyveromyces marxianus*, *Yarrowia lipolytica*, *Pichia hudriavzevii* and *Torulaspora delbrueckii*) from diverse sources, including traditional fermented foods, the human gut, and the environment [16]. The therapeutic use of probiotic yeasts derived from fermented foods for infections associated with *Candida* species has been identified as a promising area of research. Potential probiotic yeasts offer an additional method for treating gut microbiota dysbiosis and preventing health complications that arise from opportunistic fungal colonisation, especially drug-resistant superbugs [17].

Notwithstanding the aforementioned positive properties of yeasts, they are capable of thriving in a broad spectrum of environmental conditions [18], which can induce undesirable processes, e.g., a decrease in pH. In addition to the presence of authentic yeasts, the possibility of contamination by non-indigenous microbes is also present, which can cause other undesirable changes. In many cases, they are responsible for the spoilage of food, a process that is often associated with fungal activity [19]. The utilisation of food components as growth substrates by yeasts results in the transformation of these substrates into a wide array of metabolic end products. Consequently, the chemical, physical, and sensory properties of the food are subject to modification [20]. As indicated by the findings of recent studies, manufacturing facilities and equipment are considered to be the main potential sources of spoilage yeast contamination in processed foods and beverages. This phenomenon is attributable to the selective pressure exerted by the particular conditions of the plant production environment [21].

Whilst not generally associated with foodborne illnesses, it is important to note that certain yeasts have the capacity to cause infections in humans and animals. Some species of yeast, including *Candida* spp., have the capacity to enter the human body through food and beverages, thereby potentially causing various types of infections. The most common yeast-related infection is likely to be candidiasis, which is caused by diverse *Candida* species, such as *Candida albicans*, *C. tropicalis*, *C. krusei*, *C. glabrata*, *C. guilliermondii*, *C. parapsilosis*, *C. lusitaniae*, *C. kefyr*, *C. rugosa*, *C. dubliniensis*, and *C. viswanathii* [19,22]. Nevertheless, it is not only the yeast itself that can pose a threat to foodstuffs or their processing environment, but also the smaller organisms that may be concealed within them. In the study by Salmanian et al. [23], the proposition was made that foodborne yeasts, capable of surviving stressful conditions during food processing (e.g., heat and sanitisers), could function as potent reservoirs of *Helicobacter pylori*, a gastric pathogen, thereby facilitating its transmission to humans. The occurrence of yeast in food materials could be regarded as a potential indicator of bacterial contamination [23]. Consequently, the reduction of yeast content in foodstuffs through the implementation of rigorous hygienic practices during food processing may be imperative for the management of potential bacterial infections.

The objective of the present summary article, based on the aforementioned literature, is to provide a concise overview of the methodologies employed for the detection and identification of yeasts in food products. This is undertaken with the aim of assessing the relationships between food systems and yeasts, thereby optimising food production, ensuring food safety and minimising the risks associated with contamination.

## 2. Yeasts in Foods

According to the latest taxonomic knowledge, the number of yeast species is estimated to be approximately 2000–2200, with many of these being present in various food sources [24]. A survey has revealed that around 30% of yeast isolates in strain collections are of plant origin, while 6% are from fermented beverages [25]. The development of identification techniques has enabled researchers to describe new yeast species. For instance, recent research has identified new species from various foodstuffs, including olive oil [26], grapes [27] and meat [28,29]. This development is significant in facilitating the understanding of the microbiome of complex food matrices as a source of the gut microbiome. Furthermore, it presents an opportunity to enhance fermentation processes by isolating microorganisms with novel properties. Consequently, it can contribute to the quality and safety of foods [24].

Yeasts possess the capacity to adapt to a broad spectrum of conditions, including extreme environments [30]. The impact of environmental factors, such as pH, temperature and water activity, can vary significantly due to the heterogeneity of microenvironments. These factors are recognised as primary physical elements influencing microbial growth in food. The nutrient-rich nature of food provides a conducive environment for yeast growth and metabolism. However, the ability to utilise various nutrients is one of the most important ecological factors determining the habitat specificity of yeasts. Many yeasts can metabolise glucose and other simple sugars via both fermentation and the oxidative pathway, whereas other carbon sources can only be used via aerobic respiration [31].

Food microbial communities are susceptible to changes in environmental conditions, such as decreases in pH and increases in alcohol content, that occur during the production process. Furthermore, the relationships and interactions among community members can also influence the community structure itself. The growth, survival, and activity of desirable, spoilage or pathogenic organisms are determined by other species or strains [32].

Biotechnological processes are of significance in the context of food production. In these processes microbes originating from the environment and the natural microbiota of raw materials are employed to transform these materials into enriched foodstuffs. This transformation involves the modification of their chemical and physical characteristics. The complex biochemical processes carried out by microbes serve to enhance the organoleptic and nutritional properties of the food, extend its shelf-life, and contribute to its safety [33].

**Figure 1:**
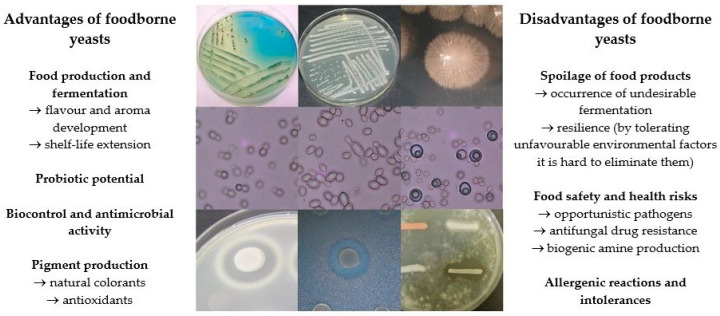
The impacts of foodborne yeasts. (The images illustrate the macro- and micromorphological diversity, as well as the enzymatic and biocontrol activity of yeasts. Own photographs.).

It is well established, that the role of yeasts in foods is twofold; they can be beneficial, but they can also cause food spoilage, resulting in significant economic loss (Figure 1). It is important to note that the presence of the same species can be beneficial or detrimental depending on the food type. A considerable amount of research, encompassing both comprehensive studies and books, has been dedicated to elucidating the role and significance of yeasts in the food industry and the species present in various foodstuffs [34,35,36,37,38,39]. Consequently, the most important aspects will be emphasised in the discussion that follows.

The domesticated yeast species *S. cerevisiae* plays a pivotal role in the fermentation process, which is utilised in the production of various alcoholic beverages, including wine, sake, and ale, a type of beer. Additionally, it is employed in the fermentation of bread and sourdough [40,41]. A metagenomic analysis of 2500 foods has revealed the high genetic variability of this species, and its specialisation for different foodstuffs [24]. In addition to *S. cerevisiae*, many of the yeast species, including a large number of NSYs, take part in the different types of fermentations to produce foods and beverages [35].

Traditional food and beverage fermentations are complex processes involving interactions between different microorganisms [42]. These interactions occur between bacteria and various fungal species, including yeasts, which are involved in the fermentation process. A broad spectrum of yeast genera has been reported in association with various fermented foods and beverages worldwide [43].

Yeasts play a pivotal role in both the fermentation and maturation processes, particularly in the context of cheese production. The most prevalent species employed in the ripening of cheese include *D. hansenii*, *Galactomyces candidus*, and *K. marxianus*. However, *Y. lipolytica* also contributes significantly to the maturation process, particularly in the case of blue cheeses [38].

Yeasts play a considerable role in the regulation of undesirable microbes. The most significant group of antagonistic fungi are the killer yeasts, which are capable of producing protein or glycoprotein toxins (zymocines) that are lethal to sensitive yeast strains [44]. Yeasts that carry these traits occur naturally in large numbers in plants and in fermenting materials [45]. These organisms can be used as a starter culture in the production of wine, aiding in the regulation of fermentation processes and the elimination of contaminating wild yeasts. Additionally, they can function as natural preservatives in food products, contributing to their longevity [46].

Spoilage is the result of microbial activity, with yeast being a primary cause of food spoilage across a wide range of food matrices [19,47]. However, a significant number of yeast species possess the capacity to induce undesirable alterations in food matrices. Of particular concern are *Zygosaccharomyces* species, including *Z. bailii* and *Z. rouxii*, which are indubitably the most problematic class of yeasts encountered in the food and drink industries [48]. Although their presence is beneficial and even necessary in the production of some oriental foods.

In the context of NSYs, also called non-conventional yeasts, *Candida* has been observed to be a prevalent organism in a variety of foodstuffs, including cheese, coffee, cocoa, vegetables, meat, and alcoholic spontaneous fermentations [49]. The utilisation of *Candida* species as an adjuvant in cheese production or as starters for coffee, cocoa, vegetable, meat, beer, and wine fermentations has been well documented. A thorough screening of potential *Candida* species is often conducted to identify the most effective strains, with the objective of enhancing specific characteristics. Notable species of *Candida* that are frequently selected include *C. zemplinina* (synonym *Starmerella bacillaris*) and *C. pulcherrima* (teleomorph *Metschnikowia pulcherrima*) (grape and wine) [50,51,52] *C. parapsilosis* (teleomorph *Monilia parapsilosis*) (coffee) [53], *C. famata* (teleomorph *Debaryomyces hansenii*) (cheese and meat) [24,38], *C. zeylanoides* (teleomorph *Kurtzmaniella zeylanoides*), and *C. norvegensis* (teleomorph *Pichia norvegensis*) (cocoa) [54]. Among these species *C. famata* has been identified as the most prevalent yeast in food products following *S. cerevisiae*, according to metagenomic analysis of foods carried out by Carlino et al. [24]. This study revealed that the prevalence of *C. famata* has been observed to be particularly significant in fermented meat (64%) and dairy products (24%), with its presence detected in 19% of the food samples examined and across seven distinct food categories.

The above-mentioned *Candida* species are associated with the production of key metabolites (food aroma formation) and different enzymes. However, safety-associated selection criteria are often neglected. It is widely recognised that certain *Candida* species pose a significant threat as opportunistic human pathogens, as they have the capacity to penetrate the human body through the consumption of food and beverages, resulting in substantial clinical implications [19,49].

## 3. Detection of Yeasts from Food Matrices

The analysis of foods for the presence of both pathogenic and spoilage bacteria is standard practice for ensuring food safety and quality. Conventional bacterial testing methods rely on specific microbiological media for the isolation and enumeration of viable bacterial cells in foods. These methods are distinguished by their sensitivity, cost-effectiveness, and ability to provide both qualitative and quantitative data on the number and nature of the microorganisms present in a food sample [55].

The mycological examination of foods often aims only to enumerate the colony-forming units (CFU) of “yeasts and moulds” together. While this information may relate to the general contamination of products, it may be meaningless or even misleading for assessing the mycological safety, quality, and stability of foods, given the fundamental differences between the two groups of fungi [56]. Consequently, the accurate detection and quantification of yeast species is imperative for ensuring the quality control of these products [18].

Culture-based detection methods are traditional microbiological techniques used to identify and quantify foodborne yeasts by growing them on culture media. Their advantages and disadvantages are listed in Table 1. The conventional culture-based methods are time-consuming, as they rely on the ability of microorganisms to multiply into visible colonies, which can take several days. Moreover, the preparation of culture media, the inoculation of plates, colony counting and biochemical characterisation are all processes that are labour-intensive. This has led to a need for more rapid methods in the food industry to provide adequate information on the possible presence of microorganisms, mainly pathogens, in raw materials and finished food products, for manufacturing process control and for the monitoring of cleaning and hygiene practices. These rapid methods address the early detection and enumeration of microorganisms, as well as the characterisation of isolates through the utilisation of a wide range of approaches, including microbiological, chemical, biochemical, biophysical, molecular biological, immunological and serological methods [55].

### 3.1. Standards for the Enumeration of Yeasts from Food, Feed and Their Changes

**Figure 2:**
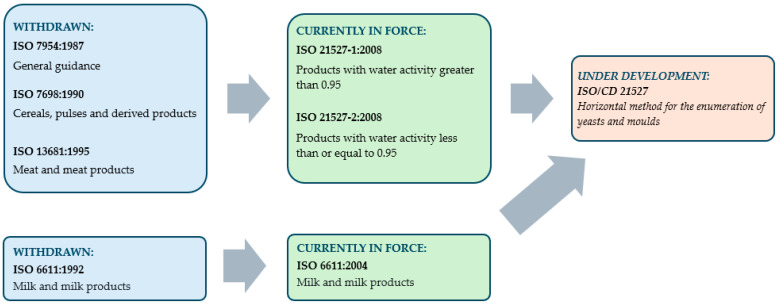
Life cycle of the evolution of standards for enumeration of viable yeasts and moulds from 1987 to the present and with a focus on the future [61,62,63,64].

The International Organization for Standardization (ISO) has published numerous standards dealing with the enumeration of yeasts and moulds in different foods and feeds over the last 40 years (Figure 2).

The ISO 7954:1987 [65] standard is a general guideline for the enumeration of yeasts and moulds for food and feeds. In the subsequent years, three distinct standards—ISO 7698:1990 [66], ISO 13681:1995 [67] and ISO 6611:1992 [68]—have been developed, providing specific guidelines for the enumeration of yeasts and moulds in various food products, including cereals, pulses, meat, and meat products, milk and milk products. One of the common features of these standards is the application of the same culturing technique—the pour-plating method. The culture media recommended by the standards is Yeast Extract Dextrose-Chloramphenicol Agar (YGC), as outlined in the general guidelines and in the standard for cereals, pulses, milk, and milk products. Chloramphenicol can be substituted by oxytetracycline. In instances where the enumeration of yeasts and moulds in meat and meat products is concerned, the utilisation of Yeast Extract Dextrose Oxytetracycline and Gentamicin Agar is recommended [65,66,67,68], as chloramphenicol or oxytetracycline proves to be inadequate in sufficiently inhibiting Gram-negative bacteria, which are prevalent in raw meat.

The aforementioned standards were withdrawn in the early 2000s, with ISO 6611:1992 being replaced by ISO 6611:2004, a document which introduced only minor changes [63]. ISO 7954:1987, ISO 7698:1990 and ISO 13681:1995 have been replaced by ISO 21527-1:2008 and ISO 21527-2:2008 [61,62,65,66,67]. The new standards categorise different food types into two groups based on their water activity, with foods falling into the categories of those with water activity greater than 0.95, or less than or equal to 0.95. In addition, it is recommended that the spread plate technique be employed for the inoculation of samples, as this method ensures higher enumerations and prevents the risk of thermal damage to microbes. Additionally, the standards in force at present demonstrate alterations in the utilisation of culture media: Dichloran Rose Bengal Chloramphenicol Agar (DRBC) is recommended for foods with water activity greater than 0.95, including but not limited to eggs, meat, most dairy products, fruits, vegetables. Conversely, Dichloran 18% Glycerol Agar (DG18) is recommended for food products with water activity less than or equal to 0.95, including but not limited to dehydrated cereals, oleaginous products, spices, seeds, powders of instant drinks [61,62].

At present a new standard—ISO/CD 21527 Microbiology of the food chain—Horizontal method for the enumeration of yeasts and moulds—is under development. According to the abstract of the future standard [64] it will be applicable to all samples belonging to the food chain. “*Because of the large variety of products in the food chain, it is possible that this horizontal method is not appropriate in every detail for all products. Nevertheless, it is expected that the required modifications are minimised so that they do not result in a significant deviation from this horizontal method*” [64].

### 3.2. Culturing Techniques and Culture Media Used for Detection of Yeasts

The selective detection of yeasts has been an area of research since the beginning of the last century. Conventional methods for quantitative and qualitative detection of yeasts include dilution plating techniques and direct plating techniques [69]. The sample preparation procedures can vary considerably depending on the nature of the food including shaking, soaking, blending of the food in diluent [69], surface disinfection of crops [70], enrichment of osmotolerant yeasts [71], use of glucose-water diluent to prevent osmotic shock [72], transfer of yeasts from olive oil into an aqueous phase by shaking and centrifuging [73].

Initially, media that inhibited bacteria but allowed fungi to grow were developed by lowering the pH of culture media of natural origin such as acidified potato dextrose agar (pH 3.5), wort agar (pH 4.8) or malt extract agar (pH 3.5) [57,69]. Subsequently, the utilisation of media for the general detection of yeasts and moulds by the incorporation of antibiotics became a prevalent practice (Table 2).

Following a comprehensive evaluation of the media employed for the detection of yeasts and moulds, it has been determined that antibiotic-supplemented media demonstrate superior performance in determining populations in the majority of food types when compared to acidified media [74,75].

**Table 2:** General purpose culture media for selective detection of yeasts and moulds in food.

Type of Culture Medium	Name of Culture Medium	Reference
Acidified culture media	Acidified Malt/Whey/Wort Agar	[76]
	Acidified Potato Dextrose Agar	[77]
	Orange Serum Agar	[78]
Antibiotic supplemented culture media	Dichloran Rose Bengal Chloramphenicol Agar	[79]
	Rose Bengal Chloramphenicol Agar	[80]
	Oxytetracycline-Glucose-Yeast Extract Agar	[81]

Selective culture media have also been developed for the enumeration of yeasts in certain food products including. Alcoholic fermented products, dairy products, and foods with low water activity (Table 3). Furthermore, it was found that yeasts specifically present in low-pH foods, such as wines and fruit juices, produce recovery numbers on acidified media that are equivalent to those recovered on antibiotic-supplemented media [82].

### 3.3. Detection of Yeasts by Non-Conventional Methods

In the field of food-borne yeast detection, conventional culture-dependent methods have been challenged by the emergence of more effective, expeditious, and straightforward techniques, as conductometry/impedimetry, ATP bioluminescens, immunological and molecular biological methods, and diverse advanced technologies. These are discussed in the subsequent subsections of this article. Examples of commercial kits used for the detection and/or identification of yeasts from diverse sources are summarised in Table 4.

#### 3.3.1. Conductometry/Impedimetry

Among the array of rapid and automated instrumentation methods that emerged in the 1970s, those reliant on the measurement of the electrical properties of the medium proved to be the most effective [105]. Impedance microbiology is based on the principle that metabolising organisms alter the chemical and ionic composition of their growth medium, thereby changing its impedance [106]. These instruments exploit the principle that microbial metabolism produces a large number of small molecules, generally ions and charged particles, which are usually better conductors than the original molecule. In the so-called direct method, the change in conductivity of the culture medium is measured and a growth curve can be drawn from the data. Similarly to that in the “real” growth curve, phases (lag, accelerating growth, exponential, reduction in growth and stationary phase) can be identified. The typical metric in these systems is the detection time or time to detection (DT or TTD), the time from inoculation to the time when a significant change from baseline can be measured. The direct method can be used to detect bacteria, and a calibration curve (x axis: logarithm of cell count versus y axis: TTD) can be used to estimate the cell count of the sample.

However, yeasts do not alter the conductivity of the nutrient solution. They normally cause only minor changes in conductance when growing in most laboratory media [107] and usually cause reduction in conductance [108]. For the detection of yeasts a method as indirect conductimetry was described by Owens et al. [109]. It is based on the CO_2_ production of the microorganism as an indication of microbial activity. The CO_2_ is absorbed in an alkaline solution and the rate of production is measured conductimetrically. This technique has been applied to detect foodborne pathogenic bacteria [110] and seems to be particularly suitable for the detection of yeasts which produce CO_2_ both aerobically and anaerobically. Deak and Beuchat [111] investigated the parameters (detection time, maximum rate of change in conductance and maximum extent of conductance change) that could have an effect on the change in conductivity. Their results showed that those parameters were dependent on the yeast species studied (*Candida glabrata*, *C. parapsilosis*, *D. hansenii*, *Pichia membranifaciens*, *Rhodotorula glutinis*, *S. cerevisiae*, *Z. bailii*), population density, temperature, pH, *a_w_* and preservative concentration. The advantage of this type of measurement is that it can be carried out directly in the food (e.g., fruit juice and juice concentrate) without the signal being affected by the product. Probably the major advantage of indirect conductimetry is that it is more sensitive than direct one, with a short DT and a higher rate of change in conductivity. There are currently several manufacturers of conductance/impedance instruments, which may vary, e.g., in the number of samples that can be measured, the temperature range, the test volume, and the possibility of an indirect test method.

The yeast *Brettanomyces bruxellensis* (anamorph *Dekkera bruxellensis*) is one of the major spoilage organisms faced by the wine industry. Concentrations as low as 10^3^ CFU/mL of this spoilage yeast have been known to cause unpalatable off-odours and flavours.

Recently, direct and indirect impedance methods have been compared for the detection of *B. bruxellensis* [112]. For *Brettanomyces* concentration above 10^4^ CFU/mL, the indirect method was more accurate overall compared to the ‘direct’ impedance. The ‘indirect’ impedance, in addition to offering faster detection times, is potentially less influenced by the type or composition of the wine analysed. It was concluded that the ‘indirect’ impedance has the potential to be an alternative option for the enumeration of yeasts in the wine industry because it offers less preparation time, high throughput, and the potential to reduce economic losses.

#### 3.3.2. ATP Bioluminescence

**Figure 3:**
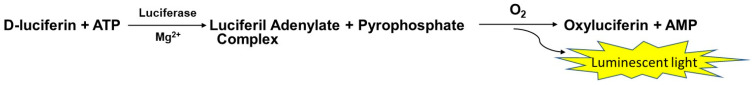
The principle of ATP luminescence.

As a result of McElroy’s research by the 1940s, it was shown that the adenosine triphosphate (ATP) is a necessary component of the reaction catalysed by the enzyme luciferase in the firefly’s light-emitting mechanism [113]. The complete reaction in the firefly luciferin-luciferase system is described in Figure 3.

In this process, the firefly (*Photinus pyralis*) luciferase enzyme catalyses the conversion of D-luciferin to oxyluciferin (Figure 3) in the presence of ATP and magnesium, while emitting yellow-green light at 560 nm [114].

The luciferase enzyme is unable to penetrate inside microbial cells and therefore, cannot access the intracellular ATP. To extract it, reagents that increase the permeability of the cell membrane are added to the cells (e.g., detergents and their mixtures, strong acids, organic solvents, etc.) or disrupt them. Chemical methods are frequently employed in conjunction with physical methods, such as short-time boiling, sonication [115] or direct current [116].

Microorganisms possess varying levels of ATP within their cells. As early as 1976, Hysert and colleagues [117] demonstrated that the ATP content of a typical yeast cell is approximately 100 times higher than that of a bacterial cell. Furthermore, the ATP content of viable microorganisms ranges from 500 to 10,000 μg per 1 g of dry biomass, which corresponds to 10^−19^ to 10^−15^ mol ATP per cell [118]. Hattori et al. [119] found that the intracellular ATP content of the yeast strains they studied differed significantly. Specifically, the ATP content was determined to be 0.714 × 10^−16^ mol/CFU for *Kluyveromyces polysporus* and 54.6 × 10^−16^ mol/CFU for *Hanseniaspora valbyensis*. In addition to yeasts, the ATP content of Gram-negative and Gram-positive bacteria was also investigated. The mean ATP content of Gram-negative and Gram-positive bacteria and yeasts was found to be 1.5 × 10^−18^, 5.5 × 10^−16^ and 8.00 × 10^−16^ mol/CFU, respectively, giving a ratio of 1:4:500 for the ATP content of these three groups of organisms.

The experimental work conducted with individual microorganisms [120] provided the basis for the development of ATP bioluminometer systems. The development of highly sensitive luminometers enabled the detection, measurement and quantification of the light produced during the reaction, utilising relative light units (RLU). The measurement process is rapid, typically requiring only a few minutes per sample. It has been established that there is a general direct proportionality between the amount of ATP in the cell and the biomass or number of cells of a given species [121]. Therefore, the amount of light produced during the bioluminescence reaction is proportional to the concentration of cells in the analysed medium under standard conditions [122].

In ATP-measuring bioluminescence systems, ATP extracted from microbial cells reacts with the luciferase enzyme and luciferin in the reagents, resulting in measurable amounts of light being generated from the ATP produced by the freshly lysed cells [123]. The amount of light produced can be amplified and read by a luminometer. Since ATP is rapidly degraded after cell death, bioluminescence measurement is a reliable tool for detecting microbial contamination. The absolute value of RLU is determined by the parameters of the instrument [124]. The light intensity recorded in RLU is proportional to the ATP concentration in the linear range of ATP between 10 fM and 1 μM [125].

Improvements in reagent quality and instrument sensitivity have led to numerous applications of bioluminescence ATP determination [123]. It is widely used in the food, biotechnology and diagnostic industries [114,123,126,127,128,129,130,131,132].

Nevertheless, it should be noted that bioluminescence testing is not a specific method and cannot distinguish between different microbial contamination sources. Consequently, selective detection of yeasts can be achieved through the implementation of either selective culturing or the utilisation of filters with distinct pore diameters, due to the difference in size between yeasts and bacteria. The majority of yeast applications are related to different areas of the beverage industry.

A rapid ATP measurement method for the bioluminescence analysis of brewer’s yeasts and bacteria was developed by Hysert and colleagues in 1976 [117]. The research team employed the method to determine the ATP content of yeast cells and medium [133] by monitoring the growth of 36 ale, lager and mixed yeasts at 12 °C, 18 °C and 23 °C in wort samples, resulting in significant differences between species. The authors observed that patterns of ATP level variation in ale and lager yeasts, especially at lower temperatures, can be used as a tool to classify and differentiate yeasts.

Miller and Galston [134] developed a series of sterility tests based on ATP bioluminescence for the detection of small numbers of yeasts in packaged beers within 1 day. The conventional cultivation method for detecting potential spoilage microorganisms in these products requires 2–3 days for yeasts, thus rendering the newly developed method highly rapid. The ATP assay was conducted following yeast incubation on selective nutrient agar for 24 h, subsequent to membrane filtration of the beer samples. In accordance with their experience, samples that exhibited more than threefold the mean RLU value of the negative control samples were designated as positive, i.e., demonstrating growth.

A yeast pitching method for beer-making was developed by researchers using ATP measurement [135]. Firefly bioluminescent ATP assay was used to quantify viable yeast cells and proved to be more consistent and suitable than the widely used conventional percent solids determination method.

In the development of a rapid assay system for beer samples [136], they were filtered through a Filtravette™ (New Horizons Diagnostics Corporation, Columbia, MD, United States) filter system (0.45 µm), which is able to separate yeasts and bacteria. The quantity of yeast recovered was determined by ATP bioluminescence measurement. The system demonstrated an effective capacity to detect 10^3^ yeasts per mL in 2 mL samples. Subsequent tests demonstrated that the sensitivity of the system could be further enhanced by increasing the sample size.

Carrick et al. [137] compared the ability of four ATP-measuring instruments to detect beer microorganisms. All instruments performed adequately with the application of liquid samples without swabs. In contrast, the four systems tested were found to have poor linearity when measuring known amounts of ATP, and ATP measurements showed high variability when swab samples containing the same concentration of microorganisms were repeated. The observations suggested that swab sampling may be the cause of the unreliability.

In addition to fermentations in the brewing industry, applications have also been made in the field of wine industry for the bioluminescence detection of yeasts. Mandl and co-workers [138] tested the suitability of ATP measurement for microbiological control of filling equipment or surfaces in cellars. Serial dilutions of the overnight cultures of *S. cerevisiae* and *Oenococcus oeni* were checked both with traditional plating and ATP measurement. It was found that the light release correlated well with the cell number values determined on the WL medium at higher yeast counts (from about 600 to 1.1 × 10^4^ CFU/mL for *S. cerevisiae* and 2.6 × 10^4^–2 × 10^8^ CFU/mL for *O. oeni*). Therefore, they concluded that the validation of the effective cleaning of filling equipment or surfaces with the evaluated device is only partially possible, as explicit ATP measurement results can only be anticipated at elevated yeast counts.

Monica and co-workers [139] inoculated wine samples with different concentrations of bacteria and yeasts. Then wine samples were filtered through two serial filters with decreasing mesh to separate bacteria from yeasts. The microorganisms on filters were resuscitated for 24 h in a selective liquid medium and analysed by bioluminescence assay. ATP measurements discriminated the presence of yeasts and bacteria in the spiked wine samples down to 50 CFU/L of yeast and 1000 CFU/L of bacteria. The developed protocol allowed rapid (24 h) and simultaneous detection of bacteria and yeasts in different wine types.

Siro and colleagues [126] have developed a continuous flow system for testing baker’s yeast to measure the ATP of yeast cells as an index of growth. They found a good correlation between the amount of cell ATP and cell growth during the exponential growth phase.

ATP bioluminescence methods are easy to perform but they require the use of specific instruments. Although some ATP bioluminescence systems allow rapid and selective detection of yeasts within a day, occasionally within an hour, they have not been widely applied in practice. The utilisation of this technique has remained limited to hygiene control.

#### 3.3.3. Immunological Methods

Immunological detection is based on antigen-antibody binding that forms so-called immune complexes. Serological methods have been employed to facilitate the rapid and specific identification of microbes, thus finding widespread application in the detection and identification of pathogens, including food-borne pathogens such as bacteria, viruses, and fungi [140]. Different types of immunological methods can be used for these purposes, such as enzyme-linked immunosorbent assay (ELISA), latex agglutination or lateral flow immunoassay [140,141].

The antigenic structure of yeasts is suitable for identification through immunological methods. Their primary antigens are cell wall-associated molecules, such as mannans and beta-D-glucans [142], but capsular polysaccharides can also serve as antigens in the case of species of *Cryptococcus* genus. Extensive research has been directed towards understanding the antigens of yeasts, including the description of the major antigens of pathogenic *Candida* species [143], *Cryptococcus neoformans* [144] *S. cerevisiae* [145], *Trichosporon* spp. [146].

A range of methods have been developed for the immunological detection of clinically significant yeasts, such as *C. albicans*, *C. krusei*, *Cryptococcus* spp. [147] and *Trichosporon* spp. Several commercially available products for their detection are currently on the market (e.g., Immuno Mycologics, Inc., Norman, OK, USA; Genobio Pharmaceutical, Tianjin, China). These include antigen and antibody tests.

Since the 1960s, several immunological methods have been developed for the detection of food spoilage yeasts [148] such as wild *S. cerevisiae* contaminating beer fermentation [149] or the yeast occurring in fermented dairy products [150].

The detection and identification of yeasts in food by serological methods has been superseded by the development of molecular methods. García et al. [150] have demonstrated that ELISA techniques using polyclonal antibodies against yeast cells are of limited value for the detection and enumeration of spoilage yeast species in dairy products, compared with the PCR amplifying 18S ribosomal DNA (rDNA) sequences.

Whilst immunological methods are generally highly specific, this specificity is dependent on the quality of the antibodies employed. Polyclonal antibodies frequently exhibit cross-reactivity, while monoclonal antibody preparations are costly and are only available for a limited number of species. Consequently, they are not well-suited for the detection or identification of a broad spectrum of yeasts, including the identification of unknown or rare species. Moreover, these methods are often less sensitive than molecular methods. Additionally, the complex nature of the food matrix can interfere with detection, necessitating specific sample preparation. The limitations of immunological methods, as outlined above, have rendered them less competitive in comparison to nucleic acid-based molecular techniques which subsequently replaced them.

#### 3.3.4. Molecular Biological Methods for Yeast Detection

In the context of the food industry, microbiological testing is of paramount importance for quality control and process compliance [151]. While presence/absence tests are qualitative methods commonly used for detecting microorganisms in food and water samples, their application specifically to foodborne yeasts is less documented. Nucleic acid-based presence/absence tests are pivotal for the rapid and accurate detection of foodborne yeasts. Molecular tests such as PCR, real-time PCR (qPCR), and DNA microarrays utilise distinct genes to detect the presence or absence of foodborne yeasts in different food matrices. A prerequisite for the implementation of these molecular tests is a comprehensive understanding of the yeast biota present in the sample, encompassing both beneficial and harmful species. The target genes are selected based on their specificity, conservation within the species of interest, and their ability to distinguish the target yeast from other microorganisms.

Commonly used target genes are the ribosomal RNA (rRNA) genes. The internal transcribed spacer (ITS1, ITS2) regions are commonly targeted for identifying and differentiating yeast species, as they exhibit interspecies variability. Yeasts were detected from sour beer [152], beer and cider made by spontaneous and mixed fermentation [153], kombucha [154], and vacuum-packed beef [155]. The 18S rRNA gene, a conserved region of the ribosomal RNA, can be used for broad-range fungal detection. Sanz et al. [156] applied this gene as a target sequence for rapid detection of yeast species in vacuum-packed ham. Moreover, the 26S rRNA gene (the D1/D2 domains) was also used for species-level differentiation of yeasts originating from dairy products [157] and detection of *Z. bailii* from fruit juices.

Among the housekeeping genes, the *TEF1* gene for elongation factor EF-1 alpha can also be used for yeast detection due to its variability among yeast species. Vaitilingom et al. [158] detected viable yeasts from contaminated milk samples after heat treatment using reverse transcriptase PCR (RT-PCR) targeting *EF-1α*. Moreover, *ACT1* (actin gene, used in many cases as a reference or control in molecular assays), *CYTB* (cytochrome b gene, which is mitochondrial DNA and can be utilised for distinguishing closely related yeast species), *COX2* (cytochrome c oxidase subunit 2 gene, another mitochondrial gene used in yeast differentiation), *CAR1* (arginase enzyme responsible for arginine degradation to ornithine and urea) and *CS1* (citrate synthase gene) are examples of alternative housekeeping and functional target genes for yeast detection from food and beverages [159,160,161,162,163].

In the context of spoilage-related genes, the *ARO* (genes encoding for aromatic aminotransferases), the *ADH* (alcohol dehydrogenase gene), and the *VPR1* (vinylphenol reductase gene liable for “Brett” sensory modification of wine) may be utilised for the detection of spoilage yeast, including *S. cerevisiae*, *M. pulcherrima*, and *Brettanomyces* species [164,165].

As demonstrated above, molecular methods offer several remarkable possibilities not only for yeast detection, but identification in addition. However, it should be noted that a number of factors have a significant impact on both the detection and quantification processes. These include the following. Firstly, the cellular copy number of the gene to be used must be taken into consideration. Secondly, it is important to ascertain whether the gene is sufficiently conserved to be PCR amplified by ‘universal’ primers that will detect all species of interest. Thirdly, the efficiency of DNA extraction from cells in the sample must be ascertained. Fourthly, the efficiency of DNA recovery from the sample must be determined. Fifthly, any sample components that may interfere with DNA recovery or PCR amplification must be identified. Finally, the level of cell population detectable must be ascertained [166].

#### 3.3.5. Application of Other Technologies in Detection of Foodborne Yeasts

Significant progress has been made in the field of methods for detecting yeasts in foods, leading to the development of novel techniques.

In recent years, novel methodologies and approaches have been pioneered for the detection of yeasts. In the study by Wu et al. [167], a multi-channel magnetic flow device was developed for the rapid separation and purification of lactic acid bacteria and yeasts. This device enabled the rapid detection of *Lactiplantibacillus plantarum*, *Lactococcus lactis* and *S. cerevisiae* as well, thus providing an effective means for dynamic real-time monitoring of lactic acid bacteria and yeast in the fermentation process. In addition, it facilitated the timely quality control of the production of fermented foods in industry.

Recently a deep learning-based approach to yeast classification was developed, integrating conventional cultivation methods, white light optical microscopy of microcolony, and deep learning techniques to facilitate rapid detection and classification of yeasts [168]. The approach has been shown to reduce the time required for yeast detection. Furthermore, the utilisation of deep convolutional neural networks enables the model to accurately discriminate between distinct yeast species within a short timeframe of six hours.

In the work of Stilman et al. [169], a novel technique for the sensitive and facile detection of *Saccharomyces* strains is published, with a low detection limit in both buffer solution and food samples, including beer and yoghurt. The detection is achieved by combining ultrathin surface-imprinted polymer (SIP) layers as receptors with impedance spectroscopy as the readout principle.

In the food industry, the formation of viable but non-culturable (VBNC) microorganisms is of significance due to the stressful conditions that prevail. Moreover, their detection is of equal importance. VBNC cells are distinguished by their inability to proliferate on conventional culture media while retaining their viability. However, they possess the potential to resuscitate under favourable conditions. This characteristic renders traditional detection methods inadequate for identifying microorganisms in the VBNC state [170]. The detection of these cells is of paramount importance, and a range of techniques are currently employed for this purpose. A pervasive approach entails the direct visualisation of live cells through the utilisation of stains and microscopic enumeration [171,172]. Salma et al. [173], Ferrario et al. [174], Xie et al. [175] and He et al. [176] used flow cytometry for VBNC yeast detection.

### 3.4. Identification and Typing of Food-Borne Yeasts

Yeasts can be identified through various methodological approaches, including the determination of phenotypic traits through the application of fermentation and growth tests, the observation of colony appearance, cell shape, and filamentation, the assessment of sexual states and mating tests, the implementation of nuclear staining techniques, and DNA-based methods for yeast identification, as outlined in the seminal work of Kurtzman et al. [177].

Nevertheless, it is important to note that alternative methods can also be utilised for the identification of yeasts isolated from foodstuffs. As demonstrated by Kümmerle et al. [178], Fourier-transform infrared spectroscopy (FT-IR) is a rapid and efficient identification method that can accurately identify yeast samples within 24 h from a single colony. This method possesses a high degree of differentiation capacity, thereby clearly surpassing other conventional methods for yeast identification. The study emphasised that FT-IR technology accurately identified 97.5% of the isolates. A rapid method for the identification of yeasts based on fatty acid profiles obtained by gas chromatography (GC) was described by Botha and Kock [179] and applied successfully by Aloklah et al. [180] for the identification of yeasts and yeast-like fungi in the case of food products.

The following sections will outline biochemical methods, various rapid tests, MALDI-TOF MS and molecular biological methods that have been deemed suitable for the identification of foodborne yeasts.

#### 3.4.1. Biochemical Identification

The biochemical identification of food-borne yeasts involves their classification and differentiation based on their metabolic and enzymatic characteristics. Commonly used methods include assessing the ability of yeasts to assimilate and ferment certain carbohydrates, as well as examining the utilisation of nitrogen sources and the production of specific enzymes In addition, to differentiate between psychrophilic, mesophilic and thermophilic yeasts, different incubation temperatures are used to determine their optimum growth range [177,181,182].

Carbohydrate assimilation tests are widely used to determine the ability of a yeast species to utilise a specific carbohydrate as its sole carbon source, generally in a basal medium such as yeast nitrogen base. Similarly, nitrate assimilation tests are used to study the ability of yeasts to utilise nitrate as the sole nitrogen source. Potassium nitrate (KNO_3_) is the only inorganic nitrogen source in this reaction, and peptone serves as a positive growth control. Carbohydrate fermentation tests are used to complement carbohydrate utilisation tests to evaluate the ability of the yeast to ferment carbohydrates, leading to the production of ethanol and carbon dioxide. Fermentation tests, however, are considered less reliable than assimilation tests due to variability in the results. The media usually contains peptone, beef extract or yeast extract and carbohydrate sources, usually glucose. For the detection of gas production, an inverted Durham tube is inserted. Due to the presence of pH indicators in the medium, colour changes can also be observed. The tubes are incubated up to 14 days before results can be obtained. Several enzymatic tests are also used for yeast identification. The most commonly used test is the urease assay. This assay is applied to differentiate between basidiomycetous and ascomycetous yeast species. Yeasts with this enzyme can hydrolyse urea into carbon dioxide and ammonia. This makes the pH of the environment more alkaline, resulting in a colour change of the medium from orange to pink or red in the presence of phenol red indicator. In the standard procedure, Christensen’s urea agar is utilised for this test; the incubation period can extend up to four to five days, with daily examinations being carried out. It should be noted that there are tests which indicate urease activity within a significantly shorter time frame—ranging from 4 to 20 h [177,183,184,185].

Certain yeasts (e.g., *C. neoformans*) produce phenoloxidase, which can produce brown or black pigment in reaction with caffeic acid. This characteristic can be detected using media containing caffeic acid [186].

Catalase tests are also used to distinguish yeast species based on their metabolic pathways. Catalase enzyme breaks down hydrogen peroxide into water and oxygen. A recent study by Bryer [187] describes a novel method of immobilising yeast into “yeast balls” to test catalase activity in hydrogen peroxide solutions.

Additionally, β-glucosidase activity is important for wine production as it helps in the hydrolysis of grape glycosides, thus influencing wine flavour and aroma. Due to the variations in β-glucosidase activity between different yeast species its presence or absence can be used for distinguishing between yeast species [188].

Chromogenic culture media such as media with fluconazole supplementation, or Brillance Candida agar provide an additional method for yeast identification based on enzyme activity, colour changes and morphological observations [189,190,191].

Commercial identification kits are also available, that include standardised, simple biochemical tests for yeast identification [192].

One of the main advantages of biochemical tests is that they are cost-effective and do not require expensive and complicated instruments, unlike molecular methods and MALDI-TOF MS. On the other hand, manual tests are labour-intensive, and require large amounts of media and long incubation periods. Additionally, biochemical tests may yield no results or misidentify unusual yeasts. Therefore, a combination of classical biochemical and molecular methods is also required for the accrue identification of food-borne yeasts, contributing to food safety [192,193,194].

#### 3.4.2. Rapid Tests for Identification

Conventional detection methods necessitate the isolation of visible yeast colonies for genetic or biochemical characterisation, a process that takes 5–7 days and requires a substantial amount of labour [168]. Conventional differentiation systems utilising morphological characteristics, in conjunction with patterns of carbon source assimilation and fermentation, are inadequate for rapid, straightforward, cost-effective identification. These systems are often laborious and time-consuming, and their efficacy is frequently restricted, as numerous species are distinguished from one another by a solitary physiological reaction, which is frequently governed by a single mutable marker [178].

The API^®^ test family developed by bioMerieux [195] comprises standardised, miniature biochemical test strips that are utilised globally for the identification of bacteria and fungi. A significant advantage of these test strips is their ease of use. The system offers a substantial and robust database accessible through an internet-based service, the APIWEB™ (BioMérieux, Marcy-l’Étoile, Lyon, France). The kits contain strips that can be used to perform up to 20 miniature biochemical tests. These tests have been developed to be quick, safe, and easy to perform. API strips are characterised by prolonged shelf life, a property that facilitates the maintenance of a constant supply of test kits within a laboratory setting.

VITEK^®^ 2 COMPACT (BioMérieux, Marcy-l’Étoile, Lyon, France) is an automated microbial identification system that provides accurate, efficient, and cost-effective phenotypic identification [195]. The features of VITEK are: a three-step setup procedure, barcoded reagents for traceability, VITEK 2 Systems Software Version 9.02 software, a comprehensive database, closed test cards to eliminate the risk of contamination from the user, and one-click result validation. The VITEK^®^ 2 YST Identification Card from bioMerieux is a diagnostic tool employed in conjunction with the VITEK^®^ 2 system to ascertain the identification of clinically significant yeast and yeast-like organisms.

BD Phoenix™ (Becton, Dickinson and Company, Mississagga, ON, Canada) is an automated system for microbial identification, susceptibility testing, and a combination of identification and susceptibility testing panels. It is designed to provide accurate and rapid results for yeast and yeast-like organisms, as well as for aerobic and facultative anaerobic Gram-negative and Gram-positive bacteria [196]. Posteraro et al. [197] tested the BD Phoenix™ system for species-level identification of 250 yeast isolates, and compared the results with the VITEK^®^ 2 system. They excluded those species not present in each system’s database, and the results revealed that BD Phoenix™ correctly identified 96.3% of the isolates, and VITEK^®^ 2 identified 91.4%. However, the BD Phoenix™ system was not able to correctly identify a rare emerging pathogen, *Kodamaea ohmeri* as this genus is not included in its identification database [198].

RapID™ YEAST PLUS System (Thermo Fisher Scientific™, Waltham, MA, USA) is a diagnostic tool to identify more than 40 different yeasts and yeast-like organisms within a few hours from clinical samples, although several studies have proved its efficacy with food-isolated yeasts as well [199,200,201]. The system combines conventional and chromogenic substrates that react with enzymes produced by yeast species, resulting in visible colour reactions. The commercial identification kits RapID™ YEAST PLUS and API 20C AUX were able to correctly identify only low numbers (35 and 13%, respectively) of the isolates [199]. Table 5 indicates some examples of the use of the above-mentioned tests for the identification of yeasts of food origin.

#### 3.4.3. Applicability of MALDI-TOF MS for Yeast Identification

##### Approach to the Methodological Concept

In recent years, matrix-assisted laser desorption/ionisation time-of-flight mass spectrometry (MALDI-TOF MS) has become increasingly prevalent in routine laboratory settings, serving as a valuable alternative for microbial identification [222]. This technique enables the measurement of peptides and other biomolecules, facilitating the analysis of complex mixtures. One of its key advantages is its effectiveness in identifying intact cells from pure cultures. The identification technique’s core principle is generating mass spectral fingerprints from ribosomal proteins, which serve as unique molecular signatures at the species level for different microorganisms. Due to its rapidity, cost-effectiveness, and capacity for high-throughput analysis, MALDI-TOF MS presents a viable alternative to traditional biochemical and molecular identification systems in laboratory diagnostics [223,224]. The sample preparation during the workflow is relatively straightforward. To elucidate the practical implementation of MALDI-TOF MS in the identification of yeast, the following steps are typically incorporated within the workflow: a single colony is selected from a culture plate and transferred to the designated target plate; one microlitre of formic acid is added with the objective of lysing the cells; following desiccation, one microlitre of HCCA matrix solution is applied; the mixture is then permitted to dry and crystallise before analysis [225]. Once prepared, the sample is subjected to short laser pulses, typically emitted from a UV/Vis laser. The matrix absorbs the laser energy, which leads to the desorption and ionisation of analytes into the gas phase. The generated ions are predominantly singly charged species due to the matrix-assisted ionisation process. An applied electrostatic field accelerates these ions, propelling them toward the detector through a flight tube under vacuum conditions. The time-of-flight (TOF) required for each ion to reach the detector is governed by its mass-to-charge ratio (*m*/*z*), where the TOF is proportional to the square root of the *m*/*z* value [226]. The resulting mass spectra provide a characteristic fingerprint of the sample, typically covering the mass range of 2000 to 20,000 *m*/*z*, where the signal-to-noise ratio remains stable and highly detectable. Since MALDI-TOF MS predominantly generates singly charged ions (z = 1), the observed *m*/*z* values correspond directly to the molecular mass of the analytes plus any cationic adducts. This high-resolution mass spectrometric analysis enables the precise identification of microorganisms and biomolecules, making it an indispensable tool in various fields of bioanalytics, clinical microbiology, and beyond [225,227].

##### Identification Reliability in the Case of Yeast

Based on the literature, identifying prokaryotic bacteria is relatively straightforward and provides reliable results—mainly due to their close correlation with 16S rRNA sequence outcomes [228]. However, the identification of eukaryotic microbes, such as yeasts and moulds, often encounters obstacles. The primary reason for this challenge is the complexity of eukaryotic cells, which is reflected in the variety of ribosomal proteins [229]. To separate them, the flight time provided by the linear TOF is sufficient only under certain conditions. Although MALDI-TOF MS is generally considered a reproducible method, variations in identification results have been reported. Several factors contribute to these inconsistencies, including differences in mass spectrometer instruments, the choice of matrix and solvent composition, the sample preparation protocol, and culture conditions such as growth medium, temperature, and colony age [230]. Moreover, strain-specific biological variability can further influence spectral profiles, complicating accurate identification. A key limitation in yeast identification is the reliance on reference databases. While commercial databases continuously expand, they lack comprehensive coverage of diverse yeast species and strains, leading to potential misidentifications [224]. The method is already employed to identify yeasts in food products [155,231,232,233]; however, its application is not exclusive to that purpose, but rather it is utilised in combination with molecular identification techniques. Some studies suggest that supplementing protein spectra with additional markers or integrating genomic data could enhance accuracy [234,235]. Additionally, species differentiation within closely related yeast groups remains an issue. For example, *Candida* and *Cryptococcus* species often display overlapping spectral profiles, requiring refined algorithms and higher-resolution spectral analysis for confident classification [225]. In clinical and industrial applications, improving sample processing techniques, such as extended protein extraction or employing specific growth conditions, has been proposed to increase identification reliability [236]. Therefore, while MALDI-TOF MS remains a valuable tool for yeast identification, its reliability depends on standardisation, database quality, and the refinement of analytical methodologies.

#### 3.4.4. Nucleic Acid-Based Identification

##### Procedure of Nucleic Acid-Based Identification Techniques

The most common method for the nucleic acid-based identification of yeasts in food is the polymerase chain reaction (PCR) technique, with the subsequent sequencing of PCR amplicons. The process of molecular identification can be categorised into four distinct phases: (i) nucleic acid isolation, (ii) PCR amplification, (iii) sequencing, and (iv) data analysis.

Two approaches are available for the extraction of the nucleic acid required for the amplification process. The analysis may be conducted following a conventional culturing technique, whereby pure cultures are employed for culture-dependent identification, or alternatively, in the form of a total nucleic acid extraction directly from the food sample, for culture-independent analysis. A notable benefit of culture-dependent identification is that the use of pure cultures allows pre-grouping and/or typing of isolates (macromorphological, micromorphological, biochemical, nucleic acid-based) [154,155,237,238,239]. In this case of pre-grouping, only one or a few representative isolates from each cluster are identified. Typing is usually applied after identification with strains belonging to a given genus or species [240,241,242,243,244]. During the process of nucleic acid extraction, cell disruption can be achieved by mechanical (e.g., glass beads), chemical (e.g., NaOH, enzymes) or physical (e.g., heat) impact [237,245,246,247]. Once the nucleic acid is released, its purification and concentration can be undertaken. The precise design of these procedures may vary from laboratory to laboratory, but in summary, they can be described as conventional nucleic acid extraction techniques. In addition, the use of various commercial kits is becoming more common, but combinations of the two methods (conventional technique–commercial kit) are more often encountered [233,248]. The use of colony PCR is rare [154].

The primary target for yeast identification is ribosomal DNA, which contains both conserved (small subunit (SSU), 5.8S and large subunit (LSU)) and more variable sequences (internal transcribed spacers (ITS1 and ITS2), D1 and D2 regions of LSU) [245,249,250]. For many years, restriction fragment length polymorphism (RFLP) analysis of the ITS1-5.8S-ITS2 region (simply known as ITS-RFLP) has been used as an exclusive method for yeast identification [251,252,253,254,255,256] but has been pushed into the background as sequencing methods have become more widespread. Nowadays, due to the widespread use of sequencing methods, it is mostly used as a co-identification method or for pre-grouping of isolates as part of culture-dependent methods [239,241,257,258,259,260,261]. In this case, the RFLP patterns obtained can be compared with those of the yeast-id.org database. Depending on the sequencing method employed, the identification process may involve the amplification of various regions, including ITS1, ITS2, ITS1-5.8S-ITS2 (ITS for short), or the D1/D2 domain (complete or partial). The primer pairs used for amplification include the originally designed primers, but several modified versions are also in use (Table 6).

Depending on the sequencing method, additional steps may be required after PCR amplification. In the case of automated Sanger sequencing, which is frequently employed, a single purification step is sufficient. Illumina sequencing, a second-generation next-generation sequencing (NGS) technology, is used to a similar extent. In this case, sequencing libraries need to be generated. The use of other NGS technologies, such as the second-generation Pyrosequencing (454 Life Sciences) and Ion Torrent or the third-generation PacBio, for yeast identification is infrequent [233,246,248,266,267].

Data analysis also depends on the sequencing technology used. In the case of Sanger sequencing, it is usually necessary to check for unidentified peaks and trim the sequenogram. For longer sequences, the sequence is determined from two directions (using forward and reverse primers), which need to be aligned. With Illumina sequencing platforms, data analysis involves several steps (trimming of primers and adapters; filtering–low-quality bases, chimeric sequences, sequences of inappropriate length; merging of paired-end reads) resulting in the creation of OTUs (Operational Taxonomic Units) or ASVs (Amplicon Sequence Variants). The processed sequences or representatives of OTUs/ASVs are identified using databases. This can be conducted using either a personal database, a published database or an online database. The most commonly used online databases for fungi include NCBI (National Center for Biotechnology Information) [268], UNITE (database and sequence management on the eukaryotic nuclear ribosomal ITS region) [269], and SILVA (small (SSU and LSU sequences) ribosomal RNA (rRNA) sequences for all three domains of life) [270], MycoBank [271] and the RPD (Ribosomal Database Project) [272] or RDP classifier [273].

The main characteristics of each phase required for nucleic acid-based culture-dependent and culture-independent yeast identification from various food samples are presented in Table 7 and Table 8. The literature search, covering the past five years, was conducted using the Science Direct database.

##### Comparison of Culture-Dependent and Culture-Independent Molecular Identification Techniques

For the molecular identification of yeast species in food, both culture-dependent and culture-independent methods are used in roughly equal proportions, and in some research both methods are applied. In many cases, the culture-dependent identification process is supplemented by pre-grouping and/or typing methods (mainly repetitive sequence-based PCR (rep-PCR), randomly amplified polymorphic DNA PCR (RAPD-PCR) and ITS-RFLP) [154,237,238,239,240,241,244,252,257,258,259,260,261,284,287]. For culture-independent methods, quantitative analysis may be important in addition to community analysis, in which case qPCR can be used to analyse copy numbers.

Analysis of the culture-dependent methods shows that after nucleic acid extraction (conventional extraction—kit application—method not specified or reference to article given only) the ITS1-5.8S-ITS2 region and the D1/D2 domain sequences are determined in approximately equal proportions (Table 7). The sequencing method employed is mentioned in only a few cases (Sanger). In many cases, the mere fact of sequencing is mentioned, or alternatively, the name of the sequencing company is given. A perusal of the company’s website reveals that both Sanger and Next-Generation Sequencing (NGS) technologies are listed among their services. The taxonomical classification is usually based on the NCBI database, but in several cases the database is not specified; only the use of the Basic Local Alignment Search Tool (BLAST) is mentioned [257,286,292].

In culture-independent methods, nucleic acid isolation is mainly performed using kits compared to culture-dependent methods. PCR is more typically used to amplify smaller regions, such as ITS1 or ITS2 regions. Also, in the case of the D1/D2 domain, it is usually not the whole domain that is amplified, but only a specific part of it. In several articles, the nomenclature of the primers utilised has been modified without altering the primer sequence. In instances where the sequence of the primer is specified, the primer used can be searched back. However, in numerous cases, the sequence is not provided, or only a reference to an article is given, where other primer names are used. Furthermore, regions amplified with the same primer pairs are often designated by different names, which calls for the implementation of a standardised terminology. In terms of sequencing methodologies, Illumina’s various platforms are the most prominent. It should be noted that when employing NGS technology, it would be advantageous to specify the enzyme used in PCR (in most cases not specified), as it is important to use high-fidelity enzymes in NGS. The taxonomic identification is mainly based on the UNITE and SILVA databases, unlike the culture-dependent identification. The identification of the species is principally based on the UNITE and SILVA databases, as opposed to culture-dependent identification [152,231,238,239,248,261,266,274,294,297,299,300,301,302,303,304,307,308,309,310,311,312,314,315,316,317].

### 3.5. Future Trends in Investigation of Foodborne Yeasts

Future trends in the field of foodborne yeast detection are increasingly focused on the application of rapid, sensitive and specific methods that can be used in real time or in the field to improve food monitoring. Among the promising techniques are molecular-based detection, biosensors, spectroscopy and imaging techniques, as well as artificial intelligence and machine learning approaches.

Raman spectroscopy (RS) is based on an optical technique that analyses the inelastic scattering of monochromatic light, primarily from laser sources, interacting with molecular vibrations in a sample [318,319,320,321]. A small fraction of the light is inelastically scattered, resulting in a shift in frequency that reveals molecular information about structure, symmetry, electronic environment, and bonding [320,321]. This wavelength shift, determined by the chemical structure of the scattering molecules, enables both qualitative and quantitative analysis of compounds and provides insights into their biochemical composition and structural characteristics [318,321,322]. The typically low-intensity spontaneous Raman signal can be amplified by Surface-Enhanced Raman Spectroscopy (SERS) when molecules are adsorbed onto the nanometallic surfaces [320,321,323], which increases the sensitivity of the technique.

RS is considered to have several advantages. Firstly, it is a fast, non-invasive technique allowing for the analysis of various sample types—solid, liquid, or gaseous—in real time and offers the possibility of in situ measurements. The method offers additional benefits, including cost efficiency and portability. In addition, it has the capacity to generate substantial quantities of data in a relatively short timeframe with minimal sample handling, as it does not necessitate the extraction or labelling of samples [318,322,323]. Furthermore, it provides precise chemical information, such as a molecular fingerprint, thus enabling comprehensive studies of both organic and inorganic compounds, including biomolecules and microorganisms [321].

Application of RS allows detection and identification of pathogens, through the precise analysis of their distinctive spectral profiles, even in complex microbial matrices [318,321]. Combined with microscopy, RS serves as a powerful tool for detecting single cells. Single-cell Raman spectroscopy (SCRS) is a highly sensitive technique with advances in pathogen diagnostics based on single-cell Raman spectra, facilitating significant progress in microbiology by allowing the analysis of single cells without the need for cultivation [319,324].

The recent extensive employment of RS for the identification of clinically significant microorganisms has rendered a convenient technique for microbial studies [321,322,323]. While it is primarily used to identify pathogenic bacteria, it has also been used to detect clinically significant fungi [321,322,324]. It is important to note that this method has yielded promising results in the case of yeasts in food, including *Saccharomyces*, *Zygosaccharomyces* and *Brettanomyces* species [325,326]. The study by Li and colleagues [320] reviewed the practical applications of RS in the assessment of foodborne pathogens and discussed the development trends and challenges of this technique in the field of food safety analysis. In conclusion, RS could become a reliable method for yeast identification in the future due to its high reproducibility, ability to obtain spectra from microcolonies, even single cells, and effective quantification.

In recent years, there has been a noticeable increase in the use of deep learning-based detection methods for fungi in scientific publications [168,327]. The “deep learning (DL) approach” to microorganism detection involves using deep neural networks (DNNs) to automatically recognise and identify microorganisms based on diverse data. This data can include microscopic images, DNA sequences, spectral signatures or growth pattern data. The primary objective of DL is to facilitate faster, more accurate, and frequently non-invasive detection. This is of particular importance in clinical or environmental settings, where rapid and accurate identification can significantly impact treatment decisions or monitoring efforts [328].

DL is a constituent element of the broader category of artificial neural networks (ANNs). The conceptualisation of ANNs was inspired by the functioning of the brain, with neurons organised in successive layers that are interconnected, extending from the input layer (e.g., images) to the output layer (e.g., bacteria presence). Through the utilisation of labelled data (e.g., images of known bacteria), these networks are able to discern patterns that enable the subsequent recognition of unseen data. The term ‘deep learning’ is used to denote ANNs that possess multiple consecutive layers. Such networks are referred to as DNNs. The advent of sophisticated computing capabilities has only recently rendered such structures feasible because previously, the computational demands of training deep neural networks made their practical use in microbiology unfeasible [318].

The utilisation of DL methodologies, employing convolutional neural networks (CNNs), has been demonstrated to be a highly efficacious approach for detecting and analysing fungal species [329]. A deep learning-based approach for the classification of yeast has recently been developed. This approach integrates conventional cultivation methods, white light optical microscopy of microcolony, and deep learning techniques. The purpose of integrating these methods is to facilitate the rapid detection, discrimination and classification of yeasts [168]. In the study of Zieliński et al. (2020) [327], a machine learning approach based on deep neural networks and bag-of-words approaches was applied to the classification of microscopic images of various fungal species. Consequently, the final stage of biochemical identification was deemed redundant, thereby reducing the identification process by 2–3 days and decreasing the cost of diagnosis. Such improvements can prove particularly valuable in resource-constrained laboratories or in outbreak scenarios requiring a rapid response.

## 4. Conclusions

The detection and identification of yeasts from foods are crucial, as it is of paramount importance to prioritise the prevention of food spoilage and the elimination of pathogenic microorganisms from food. Food and agricultural environments may act as reservoirs for resistant yeast strains, facilitating their spread.

Certain yeasts, such as *Candida*, *Pichia*, *Brettanomyces,* or *Zygosaccharomyces*, can cause food spoilage, leading to economic losses and reduced shelf life. Others, like *Cryptococcus* or *Candida auris*, may have pathogenic potential. It is becoming increasingly apparent that yeast species, like *C. auris*, are demonstrating a marked increase in prevalence, whilst concurrently exhibiting resistance to antifungal treatments. The presence of yeasts that carry antimicrobial resistance genes is a matter of concern, particularly in food-related environments, as it can lead to the transfer of these genes to other microorganisms. Nevertheless, yeasts fulfil a beneficial role in the fermentation of certain products (e.g., bread, beer, wine, and dairy products). Therefore, the monitoring and identification of these yeast species is essential to ensure consistency in fermentation and the quality of the final product. Moreover, the identification and characterisation of novel yeast isolates may result in the discovery of new starter strains that possess advantageous properties.

Nonetheless, it is imperative to emphasise the future significance of the detection and identification methods of foodborne yeasts. A substantial correlation has been identified between climate change and food production. Rising temperatures and alterations to the environment have the potential to modify microbial populations, resulting in the emergence of novel yeast species within food systems. Moreover, with the rise of plant-based and novel foods such as lab-grown meat and algae-based products (so-called next-generation food products) the role of yeasts in fermentation and contamination will become more significant. Thus, the development of rapid molecular methods (e.g., metagenomics, real-time PCR, whole-genome sequencing) will enable faster and more accurate identification of yeast species in food.

The aforementioned facts underscore the vital importance of the early detection of foodborne yeasts. A comprehensive understanding of yeast ecology in food chains is imperative to mitigate health risks and develop improved food safety strategies.

## Figures and Tables

**Table 1:** The merits and demerits of culture-based detection of foodborne yeasts based on [57,58,59,60].

Advantages	Disadvantages or Limitations
Viability Assessment: only live, viable yeasts will grow on culture media, allowing for an accurate assessment of contamination risks	Low sensitivity: may result in the failure to detect viable but non-culturable (VBNC) yeasts, or those present in low numbers
Cost-effective: relatively inexpensive in comparison to molecular methods, due to the relatively low cost of equipment and reagents, and the requirement of only rudimentary laboratory facilities	Time-consuming: requires incubation periods that can extend from 24 to 72 h or more, thereby delaying the delivery of results
Isolation and characterisation: enables isolation of pure cultures for further phenotypic and genotypic characterisation (e.g., morphology, biochemical tests, antifungal susceptibility)	Lack of specificity: morphologically similar colonies may not be distinguishable without further testing or molecular confirmation
Quantification: colony counts (e.g., CFU/mL or CFU/g) provide quantitative data on levels of yeast contamination	Selective media limitations: some yeast strains being susceptible to inhibition by the selective agents or exhibiting impaired growth on standard media
Simplicity and accessibility: straightforward protocols that are widely available and do not require specialised expertise	Labor-intensive: requires the implementation of manual plating, the undertaking of incubation, and the execution of colony counting, therefore these procedures can be arduous and prone to human error
Standardized protocols: established international guidelines for common food matrices, e.g., in the form of ISO methods, which serve to ensure the standardisation of protocols	Underestimation in mixed populations: in heterogeneous population structures, the proliferation of faster-growing or more robust yeast strains may result in the underestimation of diversity or abundance

**Table 3:** Culture media used for selective detection of yeast from special food origin.

Food Type/Occurrence	Detected Yeast(s)	Culture Medium	Selective-Differential Factors ^a^	References
Beer	most culture yeasts, nonculture yeasts and Gram-negative bacteria commonly found in breweries	Universal Beer Agar	cycloheximide	[83,84]
	most culture yeasts, nonculture yeasts and Gram-negative bacteria commonly found in breweries	Brewers’ Tomato Juice Medium	cycloheximide	
	wild yeasts—other yeast genera than *Saccharomyces*	Lysine Agar	lysine as sole nitrogen source	[85]
	wild yeasts (*Saccharomyces pastorianus*)	Schwarz Differential Medium (SDM)	Fuchsin–sulphite mixture	[86]
	inhibits, or markedly restricts, growth of brewery culture yeasts while permitting growth of many wild yeasts	Lin’s Wild Yeast Differential Agar (LWYD)	chrystal violet	[83]
Alcoholic fermentation beverages (wine, cider)	enumerating *Saccharomyces* during the early stages of fermentation	Ethanol Sulphite Yeast Extract (ESY) agar	ethanol, sodium metabisulfite	[87]
	monitoring growth of *Kloeckera apiculata*, *Saccharomycodes ludwigii*, and *Candida stellata*	Lysine Agar	lysine as sole nitrogen source	[88]
	*Brettanomyces*	*Brettanomyces* Selective Medium (BSM)	cycloheximide, chloramphenicol, gentamicin, chlorotetracycline	[89]
		*Dekkera/Brettanomyces* Differential Medium (DBDM)	cycloheximide, ethanol, p-coumaric acid, bromocresol green	
		*Brettanomyces/Dekkera* specific medium (DHSA)	cycloheximide, penicillin, gentamicin, ethanol, trehalose, saccharose, sorbic acid, bromocresol green, nutrients	[90]
		Wallerstein Differential Agar (WLD Agar, WL Differential Agar)	cycloheximide, bromocresol green	[91]
Blue-veined cheese	isolation of yeasts	Dichloran Rose Bengal Chloramphenicol Agar (DRBC)	dichloran, chloramphenicol	[92]
		Rose Bengal Chloramphenicol Agar (RBC)	chloramphenicol	
Koumiss (spontaneously fermented mare’s milk drink)	native populations of *Saccharomyces*	Ethanol Sulphite Agar supplemented with antibiotic	ethanol, sodium metabisulfite, chloramphenicol	[93]
Ogi, mawè, gowé and tchoukoutou (traditional African fermented food products)	*Candida krusei*, *Clavispora lusitaniae*, *Saccharomyces cerevisiea*, *Candida tropicalis*, *Kluyveromyces marxianus*, *Candida rugosa*	Malt extract Yeast extract Glucose Pepton Agar (MYGP) supplemented with antibiotics	chloramphenicol and chlortetracycline	[94]
Part-skim mozzarella cheese	yeast detection	Potato dextrose agar (PDA) supplemented with antibiotic,	chlortetracycline,	[95]
		Dichloran Rose Bengal Chloramphenicol Agar (DRBC)	dichloran, chloramphenicol	
Serpa (Portuguese cheese)	yeast detection	Rose Bengal Chloramphenicol Agar (RBC)	chloramphenicol	[96]
Spoiled olive oil	*Brettanomyces acidodurans*	Rose Bengal Chloramphenicol Agar (RBC)	chloramphenicol	[73]
Tropical fruits	*Saccharomyces*, *Debaryomyces*, *Rhodotorula* and *Cryptococcus* species	Modified Molybdate agar	the medium to contain 0.125% calcium propionate and 3% agar	[97]
Preservative sensitive yeasts	*Zygosaccharomyces bailii* and, to a lesser extent, *Z. rouxii*	malt acetic acid agar	standard malt extract agar to which 0.5% acetic acid is added just before pouring	[98]
Osmophilic yeasts	*Zygosaccharomyces rouxii*, *Debaryomyces hansenii*	Dichloran 18% Glycerol Agar (DG18)	dichloran, chloramphenicol, glycerol	[99,100]
	*Zygosaccharomyces bailii* in wine	*Zygosaccharomyces bailii* Differential Medium (ZBD), *Zygosaccharomyces bailii* Selective Agar (ZBA)	mineral medium supplemented with vitamins, oligoelements, formic acid-glucose mixture, pH 4.5	[101]
	*Zygosaccharomyces rouxii* in high-sugar products	Yeast Extract Glucosa Agar (YEG50 Agar)	glucose (50%) a_w_ 0.909, pH 4.5	[71]
	osmophilic yeast count in grape juice	Malt Extract Yeast Extract Glucose (50%) Agar (MY50G)	glucose (50%)	[72]
	*Zygosaccharomyces bailii* from spoiled commercially processed food	Acidified Yeast Extract Malt Agar (AYMA)	pH 3.8	[102]
		Acidified Tryptone Glucose Yeast Extract Agar (TGYA)	pH 3.9	
		modified *Zygosaccharomyces bailii* Selective Agar (ZBA)	pH 4.0	
	*Schizosaccharomyces osmophilus* in bee bread	Peptone Yeast extract Glucose and Fructose medium	glucose 5% (*w*/*w*) and fructose 55% (*w*/*w*)	[103]
	*Zygosaccharomyces favi* in bee bread	Rose Bengal Chloramphenicol Agar (RBC)	chloramphenicol	[104]
		50% Glucose Agar	glucose (50%)	

^a^ Selective-differential factors create an environment conducive to the selective detection of yeasts. Such factors include compounds that inhibit bacteria (e.g., antibiotics), components that lower the pH of the culture medium or components supporting the growth of yeasts of special demands (e.g., high sugar concentration).

**Table 4:** Commercial kits for rapid detection and/or identification of yeasts from food, beverages, and other habitats.

Name of the Kit	Application Field	Principle of Mode of Action	Target Microorganisms	Time Requirement	Producer
Auxa*Color* 2 Kit	identification	sugar assimilation	33 yeast species (most frequently isolated in human medical mycology)	48–72 h	BioRad
BioLumix Rapid Simplified Assay with YM vials	detection	CO_2_ absorption	yeasts and moulds	6–48 h	Neogen
BioTrace 4250 and BacTrac 4300	detection	impedance	yeasts and moulds, osmotolerant yeasts	within hours	Sy-Lab
D-COUNT^®^	detection	flow cytometry	yeast detection in fruit preparations, fruit juices and beverages yeast and moulds detection in non-filterable beverages and fruit juice yeast detection in yoghurts and fermented milk products	90 min	BioMérieux
Foodproof^®^ Spoilage Yeast Detection 1 LyoKit	detection and quantification	real-time PCR technology	major beverage spoilage yeasts (*Dekkera*/*Brettanomyces*, *Zygosaccharomyces* and *Saccharomyces* species)	5 h	Hygiena
HybriScan^®^ test	detection and identification	sandwich hybridisation	yeasts in foods, beverages, and water	<3 h	Merck
QuickGEN PCR Kit Yeast	quantitative detection	real-time PCR technology	contaminating yeasts from beverages	not specified	Gen-IAL
RapID™ YEAST PLUS System	identification	enzyme technology	40 different yeasts	4 h	Thermo Fisher Scientific
SCANRDI^®^	detection	combination of universal cell labelling and solid-phase cytometry	yeasts and moulds– both in vegetative and sporulated forms	less than 4 h	BioMérieux
VIT^®^ Spoilage Yeasts	detection and differentiation	gene probe technology	obligate and potentially fermentative yeasts	<3 h	Vermicon
VITEK^®^ MS and VITEK^®^ MS PRIME	identification	Matrix-Assisted Laser Desorption Ionisation-Time of Flight (MALDI-TOF) and Mass Spectrometry	yeasts and fungi from food and beverage	within a few minutes	BioMérieux
Yeast HCP ELISA Kits	detection	ELISA	*Pichia pastoris* and *Saccharomyces cerevisiae*	1 h 45 min–3 h 50 min	Sygnus Technologies

**Table 5:** Examples illustrate the application of rapid, biochemical-based tests in the identification of foodborne yeasts.

Identification Method	Source of Yeast	Reference
API 20C AUX	citrus juice	[199]
	commercial shell eggs	[202]
	Egyptian Karish cheese	[203]
	condensed milk	[204]
	fermented milk	[205]
	robusta coffee	[206]
	kefir	[207]
API 20C	partially and fully processed fruits and vegetables	[208]
	fresh sweet corn	[209]
ID32C	sour fermented foods and fodders	[210]
	traditional French cheese (“Tomme d’orchies”)	[211]
	traditional Bulgarian cereal-based beverage (boza)	[212]
	lager breweries	[213]
	commercial shell eggs	[202]
	fermented and non-fermented home-made carrot juice, irradiated radish sprout, homogenized black currant, French-type soft cheese	[214]
	seafood, beef, sushi, raw chicken	[215]
	fermented fish	[216]
	cheese	[217]
RapID Yeast Plus system	citrus juice	[199]
	Indonesian traditional fermented buffalo milk	[201]
	yoghurt	[200]
VITEK 2 system with ID-YST database	traditional Turkish fermented sausage	[218]
	medombae (rice wine)	[219]
	goat milk	[220]
YeastIdent-Food kit/ProleFood system	foodborne yeasts	[221]
Phoenix Yeast ID	carrot, parsley	[215]

**Table 6:** Most commonly used primers for rDNA amplification.

rDNA	Primer	Feature	Sequence (5′-3′)	Reference
SSU	BITS	forward	ACCTGCGGARGGATCA	[262]
	ITS1	forward	TCCGTAGGTGAACCTGCGG	[249]
	ITS1-F	forward	CTTGGTCATTTAGAGGAAGTAA	[263]
	ITS1-F_KY02	forward	TAGAGGAAGTAAAAGTCGTAA	[264]
	ITS5	forward	GGAAGTAAAAGTCGTAACAAGG	[249]
5.8S	B58S3	reverse	GAGATCCRTTGYTRAAAGTT	[262]
	ITS2	reverse	GCTGCGTTCTTCATCGATGC	[249]
	ITS2_KY02	reverse degenerate	TTYRCTRCGTTCTTCATC	[264]
	ITS3	forward	GCATCGATGAAGAACGCAGC	[249]
	ITS3_KY02	forward degenerate	GATGAAGAACGYAGYRAA	[264]
LSU	ITS4	reverse	TCCTCCGCTTATTGATATGC	[249]
	ITS4_KY01	reverse degenerate	TCCTCCGCTTWTTGWTWTGC	[264]
	NL1	forward	GCATATCAATAAGCGGAGGAAAAG	[250]
	NL4	reverse	GGTCCGTGTTTCAAGACGG	[250]
	LS2	reverse	ATTCCCAAACAACTCGACTC	[245]
	LS2-MF	forward	GAGTCGAGTTGTTTGGGAAT	[265]

**Table 7:** Culture-dependent molecular yeast identification techniques published between 2020 and 2024.

Year	Food	DNA Extraction	PCR: Amplified Region (Used Primer Pair: Forward-Reverse)	Sequencing Technique	Identification Database	Pre-Grouping and/or Typing Techniques	Reference
2024	fruit and vegetable biowastes	ns-ref ^a^	ITS1-5.8S-ITS2 region (ITS1-ITS4)	Sanger	NCBI database, MycoBank database	np ^b^	[248]
	musts and grapes	conventional nucleic acid extraction techniques	ITS1-5.8S-ITS2 region (ITS1-ITS4)	Sanger	Basic Local Alignment Search Tool (wnm ^c^)	Pre-grouping: ITS-RFLP	[257]
	cheese	conventional nucleic acid extraction techniques	ITS1-5.8S-ITS2 region (ITS1-ITS4)	Sanger	NCBI database	Pre-grouping: RAPD-PCR (primers: P1, P2)	[237]
	irradiated ready-to-eat chicken feet	TIANcombi DNA Lyse & Det PCR Kit (Tiangen Biotechnology Co., Ltd.)	ITS1-5.8S-ITS2 region (ITS1-ITS4)	scs ^d^	NCBI database	np	[274]
	boza	EasyPure^®^ Genomic DNA Kit (TransGen Biotech Co., Ltd.)	26S rDNA gene (F63-LR3)	ns ^e^	NCBI database	np	[275]
	sourdough	Wizard^®^ Genomic DNA Purification Kit (Promega Corp.)	D1/D2 domain (NL1-NL4)	scs	NCBI database	np	[276]
	baijiu	ALFA-Soil DNA Extraction Mini Kit (Guangzhou Cellgene Biotechnology Co., Ltd.)	ITS2 (ITS2-ITS3)	Illumina Nova6000 platform	NCBI database	np	[277]
	fermented milk	Fungal Genomic DNA Extraction Kit (Tiangen Biotechnology Co., Ltd.)	D1/D2 domain (NL1-NL4)	scs	NCBI database	np	[278]
	beer, cider	conventional nucleic acid extraction techniques	ITS (ns-ref)	Genetic Analyser (ABI 3130xl)	NCBI database, MycoBank database	np	[279]
	table olives	conventional nucleic acid extraction techniques	D1/D2 domain (NL1-NL4)	scs	NCBI database	Pre-grouping: rep-PCR (primer: (GTG)_5_)	[238]
2023	black olives	ns-ref	ITS1-5.8S-ITS2 region (ITS1-ITS4)	scs	NCBI database	Pre-grouping: rep-PCR (primer: (GTG)_5_); ITS-RFLP	[239]
	beer	ns-ref	ITS1-5.8S-ITS2 region (ITS1-ITS4); D1/D2 domain (NL1-NL4)	scs	NCBI database	Interspecies hybrids identification: PCR-RFLP (analysed genes: *GSY1*, *UBP7*, *MAG2*) (*Saccharomyces* sp.); ITS-RFLP (*Meyerozyma* sp.) Typing: mtDNA-RFLP (*Saccharomyces* sp.)	[240]
	grape	ns-ref	ITS1-5.8S-ITS2 region (ITS1-ITS4)	scs	NCBI database	np	[280]
	grape	ns-ref	D1-D2 loop (ns-ref)	scs	NCBI database	Pre-grouping: ITS-RFLP Typing: rep-PCR (primer: (GTG)_5_); Interdelta analysis (δ-PCR) (*S. cerevisiae*)	[241]
	grape	ns	D1/D2 domain (NL1-NL4)	Sanger	NCBI database	np	[281]
	cow-milk bryndza cheese	ns-ref	ITS1-5.8S-ITS2 region (ITS1-ITS4) D1/D2 domain (NL1-NL4)	ns	NCBI database	Interspecies diversity: MLST analysis (analysed genes: *ALA1*, *CDC19*, *GLN4*, *PGI1, PGM2*, *YSP3*) (*G. candidus*)	[242]
	Sherry wine	conventional nucleic acid extraction techniques	ITS1 and ITS2 regions, ITS5 region, D1/D2 domain (ns-ref, provided in the original article)	scs	NCBI database	np	[247]
	pellicle-forming radish paocai	Fungal DNA Extraction Kit (Tsingke Biotechnology Co., Ltd.)	ITS1-5.8S-ITS2 region (ITS1-ITS4) D1/D2 domain (NL1-NL4)	scs	NCBI database	np	[282]
2022	kombucha	MasterPure™ Yeast DNA Purification Kit (Biosearch Technologies)	D1/D2 domain (NL1-NL4)	scs	NCBI database	Pre-grouping: ITS1-5.8S-ITS2 region size; ITS-RFLP (*Saccharomyces* sp.)	[154]
	sourdough	conventional nucleic acid extraction techniques	ITS1-5.8S-ITS2 region (ITS1-ITS4)	scs	NCBI database	Pre-grouping: Start Codon Targeted (SCoT) Polymorphism marker system	[283]
	olive brine	Fungi/Yeast Genomic DNA Isolation Kit (Norgen Biotek Corp.)	D1/D2 domain (NL1-NL4)	ns	NCBI database	Pre-grouping: ITS-RFLP	[258]
	orange juice	conventional nucleic acid extraction techniques	D1/D2 domain (NL1-NL4)	Genetic Analyzer (ABI 3130)	NCBI database	Pre-grouping: PCR fingerprinting (primers: (GTG)_5_, M13)	[284]
	grape; must	ns-ref	ITS-RFLP; D1/D2 domain (ns-ref)	ns (D1/D2)	ns	Typing: Interdelta analysis (δ-PCR) (*S. cerevisiae*)	[243]
	soy sauce	ns-ref	ITS-RFLP; D1/D2 domain (NL1-NL4)	ns (D1/D2)	NCBI database	np	[285]
	fermenting cocoa beans	conventional nucleic acid extraction techniques	ITS1-5.8S-ITS2 region (ITS1-ITS4) *ACT1* gene (CA1-CA5r; CA21-CA22r; Act1-f-CA22r) D1/D2 domain (NL1-NL4)	scs	Basic Local Alignment Search Tool (wnm) Ribosomal Database Project (RDP)	np	[286]
	ganjang	conventional nucleic acid extraction techniques	whole-genome sequencing	PacBio Sequel SMRT sequencer	Pairwise sequence alignment tool LAST (lastal, ver. 1060)	np	[246]
	olive	conventional nucleic acid extraction techniques	D1/D2 domain (ns-ref)	ns	NCBI database	Pre-grouping: RAPD-PCR (primer: M13)	[287]
	mix bases for ice cream	ns	D1/D2 domain (NL1-NL4)	scs	NCBI database	Pre-grouping: DGGE analysis of D1 domain (NL1-LS2)	[288]
	coffee fermentation	ns-ref	ITS1-5.8S-ITS2 region (ITS1-ITS4)	scs	NCBI database	np	[232]
2021	mould-matured Turkish cheese	UltraClean™ microbial DNA isolation kit (Mo Bio Laboratories)	ITS1-5.8S-ITS2 region (ITS1-ITS4)	scs	NCBI database	Typing: rep-PCR (ns)	[244]
	mango	ns	ITS1-5.8S-ITS2 region (ITS1-ITS4)	ns	NCBI database	np	[289]
	olives	conventional nucleic acid extraction techniques	D1/D2 domain (ns-ref)	ns	ns	np	[290]
	fermented honey by-products	InstaGene™ Matrix (Bio-Rad Laboratories)	D1/D2 domain (ns-ref)	ns	NCBI database	Pre-grouping: ITS-RFLP Typing: RAPD-PCR (primers: M13, P80); Tandem Repeat tRNA (TRtRNA) PCR	[259]
	honey	conventional nucleic acid extraction techniques	D1/D2 domain (NL1-NL4)	Genetic Analyzer (3500 series)	NCBI database	np	[291]
	cocoa	conventional nucleic acid extraction techniques	D1/D2 domain (NL1-LS2)	scs	NCBI database	Typing: ITS-RFLP	[260]
	Vino cotto	ns-ref	ITS-RFLP; D1/D2 (NL1-NL4)	scs	NCBI database, Ribosomal Database Project (RDP)	Typing: RAPD-PCR (primer: M13)	[252]
	red wines ageing in oak barrels	conventional nucleic acid extraction techniques	ITS-RFLP; ITS1-5.8S-ITS2 region (ITS1-ITS4)	ns (*B. bruxellensis*)	yeast-id.org database	Typing: Intron Splice Site PCR (ISS-PCR); Microsatellite analysis (ns-ref)	[253]
	cheese	ns	D1/D2 domain (NL1-NL4)	ns	NCBI database, SILVA database	np	[231]
	Tibetan kefir grains	TIANamp Yeast DNA Kit (Tiangen Biotechnology Co., Ltd.)	D1/D2 domain (NL1-NL4)	Sanger	Basic Local Alignment Search Tool (wnm)	np	[292]
2020	chicha	conventional nucleic acid extraction techniques	ITS-RFLP	non-sequencing based method	yeast-id.org database	Typing: mtDNA-RFLP; Interdelta analysis (δ-PCR) (*S. cerevisiae*); Fingerprint analysis (primer: TdPIR3) (*T. delbrueckii*)	[254]
			D1/D2 region (NL1-NL4)	Sanger	NCBI database		
	tequila (fermentation)	conventional nucleic acid extraction techniques	ITS-RFLP	non-sequencing based method	yeast-id.org database, Ribosomal Database Project (RDP)	np	[255]
			D1/D2 region (NL1-NL4)	ns	NCBI database		
	Sherry wines	conventional nucleic acid extraction techniques	ITS-RFLP	non-sequencing based method	yeast-id.org database	Typing: Microsatellite multiplex PCR (analysed loci: SC8132X, YOR267C, SCPTSY7) (flor genotypes belonging to *S. cerevisiae*)	[256]
			ITS1-5.8S-ITS2 region (ITS1-ITS4)	EZ-sequencing	EMBL database, CBS database		
	grape must fermentations	ns-ref	ITS1-5.8S-ITS2 region (ITS1-ITS4)	scs	NCBI database	np	[293]
	laphet	conventional nucleic acid extraction techniques	ITS1-5.8S-ITS2 region (ITS1-ITS4)	ns	NCBI database, Ribosomal Database Project (RDP)	Pre-grouping: ITS-RFLP Typing: RAPD (primers: M13, P80); Tandem Repeat tRNA (TRtRNA) PCR	[261]
	coffee beans	conventional nucleic acid extraction techniques	ITS1-5.8S-ITS2 region (ITS1-ITS4)	scs	NCBI database	np	[294]
	sourdoughs	Wizard^®^ Genomic DNA Purification Kit (Promega Corp.)	D1/D2 domain (NL1-NL4)	Genetic Analyzer (ABI 3730)	NCBI database	np	[295]

^a^ ns-ref: not specified in the article, but reference is provided; ^b^ np: not performed; ^c^ wnm: the website (e.g., NCBI) was not mentioned; ^d^ scs: the sequencing company is specified only; ^e^ ns: not specified in the article.

**Table 8:** Culture-independent molecular yeast identification techniques published between 2020 and 2024.

Year	Food	DNA Extraction	PCR: Amplified Region (Used Primer Pair: Forward-Reverse); Used Enzyme/Kit (If Specified)	Sequencing Technique	Identification Database	Reference
2024	fruit and vegetable biowastes	Combination of conventional nucleic acid extraction techniques and QIAamp DNA Stool Mini Kit (Qiagen)	ITS2 (ITS2f-ITS2r) (Modified primers possibly developed by the authors); Advantage Polymerase Mix (Clontech) (Modified primers. In this study?)	454 Life Sciences	UNITE database	[248]
	cheese	E.Z.N.A.^®^ Soil DNA Kit (Omega Bio-tek)	D1/D2 domain (ns-ref ^a^)	Illumina MiSeq	internal database for fungi [265]; Basic Local Alignment Search Tool	[296]
	irradiated ready-to-eat chicken feet	MagAttract PowerSoil Pro DNA Kit (Qiagen)	ITS1 region (ITS1-F-ITS2) (The primer names given in the article have been corrected based on the sequence provided.)	ns ^b^	RDP classifier; Unite database	[274]
	daqu (indispensable starter)	E.Z.N.A.^®^ Soil DNA Kit (Omega Bio-tek)	ITS1 region (ITS5-ITS1) (Both specified primers are forward primers. The sequence of primers is not given.)	Illumina NovaSeq	UNITE database	[297]
	Baijiu	E.Z.N.A.^®^ Soil DNA Kit (Omega Bio-tek)	ITS1 region (ITS1-ITS2)	Illumina MiSeq	ns	[298]
	green table olive	PowerFood™ Microbial DNA Isolation Kit (MoBio)	ITS1 region (ITS1-F_KYO2-ITS2_KYO2); KAPA HiFi HotStart ReadyMix (Roche)	Illumina MiSeq	UNITE database	[299]
	natural whey starters (used in cheese making)	DNeasy PowerFood Microbial Kit (Qiagen)	ITS2 region (ITS3-ITS4_KYO1) (The forward primer name given in the article has been corrected based on the sequence provided.); *Taq* polymerase (Q Biogene)	Illumina MiSeq	UNITE database	[300]
	grape berry and juice	DNeasy PowerSoil Kit (Qiagen)	ITS1 (ns)	lllumina MiSeq	UNITE database	[301]
	table olives	MasterPure™ Complete DNA and RNA Purification Kit (Biosearch Technologies)	D1 domain (LS2-MF-NL4) (The reverse primer name given in the article has been corrected based on the sequence provided.)	lllumina MiSeq	NCBI database, SILVA database	[238]
2023	black olives	ns-ref	D1/D2 domain (LS2 and NL4MS) (LS2 is a reverse primer. NL4 is a reverse primer too. In the referred article there is no NL4MS primer.)	Illumina MiSeq	NCBI database, SILVA database	[239]
	coffee fermentation	DNeasy PowerLyzer PowerSoil Kit (Qiagen)	ITS2 region (ITS3F-ITS4R) (The primer sequences are not given, no reference is provided. Probably ITS3 and ITS4 primers.)	Illumina MiSeq	UNITE database	[302]
	kombucha	E.Z.N.A.^®^ Soil DNA Kit (Omega Bio-tek)	ITS1 region (ITS5-ITS2); Fast *Pfu* DNA Polymerase	Illlumina NovaSeq	UNITE database	[303]
	baijiu	FastDNA™ SPIN Kit for Soil (MP Biomedical)	ITS region (ITS4-ITS9) (ITS9 primer sequence or reference is not provided. It is not clear which region was exactly amplified.); DNA polymerase (TransGen Biotech)	PacBio	UNITE database	[266]
	lambic beer	Combination of conventional nucleic acid extraction techniques and DNeasy Blood and Tissue Kit (Qiagen)	ns-ref	Ion Torrent	in-house database containing a representative genome sequence	[233]
	hongqu rice wines	FastDNA™ SPIN Kit for Soil (MP Biomedical)	ITS-5.8S rRNA gene (ITS-F-ITS-R) (The primer sequence is given, but no reference. Not clear whether their own primer was developed or not.)	PacBio	ns	[267]
	caciofiore cheese	E.Z.N.A.^®^ Soil DNA Kit (Omega Bio-tek)	D1/D2 region (ns-ref)	Illumina MiSeq	SILVA database, Basic Local Alignment Search Tool	[304]
	ciauscolo PGI salami	Quick-RNA Miniprep Kit (Zymo Research)	D1 domain (ns-ref)	Illumina MiSeq	internal database for fungi [265]; NCBI database	[305]
2022	kombucha	DNeasy PowerSoil Kit (Qiagen)	D1/D2 domain (NL1-LS2); PCR-DGGE (separation, extraction reamplification without GC clamp)	scm	NCBI database	[154]
	different cheeses	Combination of conventional nucleic acid extraction techniques and DNeasy Blood and Tissue Kit (Qiagen)	D1/D2 domain (NL1-NL4)	Sanger	NCBI database	[306]
	grape; must	MasterPure™ Complete DNA and RNA Purification Kit (Biosearch Technologies)	D1 domain (ns-ref); Kapa HiFi HotStart ReadyMix (Roche)	Illumina MiSeq	internal database for fungi [265]	[243]
	soy sauce	ns-ref	ITS region (ns)	Illumina HiSeq	ns	[285]
	olive	DNeasy PowerSoil Kit (Qiagen)	ITS2 region (ITS3-ITS4); AccuPrime™ *Taq* DNA polymerase system (Invitrogen)	Illumina MiSeq	ns	[287]
	coffee fermentation	QIAamp DNA Mini Kit (Qiagen)	ITS1 region (ITS1-ITS2)	Illumina MiSeq	ns	[232]
	table olive	PowerFood™ Microbial DNA Isolation Kit (MoBio)	ITS1 region (ITS1-F_KYO2-ITS2_KYO2); KAPA HiFi HotStart ReadyMix (Roche)	Illumina MiSeq	UNITE database	[307]
	huangshui (a byproduct of baijiu fermentation)	E.Z.N.A.^®^ Soil DNA Kit (Omega Bio-tek)	ITS1 region (ITS5-ITS2) (The primer names given in the article have been corrected based on the sequence provided.)	Illumina NovaSeq	UNITE database	[308]
	grape	E.Z.N.A.^®^ Soil DNA Kit (Omega Bio-tek)	D1/D2 domain (LS2-MF-NL4) (The reverse primer name given in the article has been corrected based on the sequence provided.)	Illumina MiSeq	SILVA database, internal 26S database for fungi [265], Basic Local Alignment Search Tool	[309]
2021	grape, must	FastDNA™ SPIN Kit for Soil (MP Biomedical)	ITS1 region (ITS1-F-ITS2); FastStart Master Mix (Roche)	Illumina MiSeq	UNITE database; Ribosomal Database Project (RDP), NCBI database	[310]
	beer, cider	DNeasy PowerLyzer Microbial Kit (Qiagen)	ITS1 region (BITS-B58S3)	Illumina MiSeq	NCBI database	[153]
	cocoa	DNeasy PowerLyzer PowerSoil (Qiagen)	ITS2 region (ITS3-ITS4) (The primer names given in the article have been corrected based on the sequence provided.)	Illumina MiSeq	UNITE database	[311]
	cheese	DNeasy Blood and Tissue Kit (Qiagen) with a supplementary initial enzymatic lysis	ITS2 region (ITS3f-ITS4_Kyo1) (The primer sequences are not given. In the referred article there is no ITS3f primer, only ITS3.)	Illumina MiSeq	UNITE database	[231]
	ready-to-eat pineapple	conventional nucleic acid extraction techniques	ITS2 region (ITS3- ITS4); AccuPrime™ Taq DNA polymerase system (Invitrogen)	Illumina MiSeq	UNITE database, Basic Local Alignment Search Tool	[312]
	Tibetan kefir grains	PowerFood™ Microbial DNA Isolation Kit (MoBio)	ITS1 region (ITS1-ITS2) (The primer names given in the article have been corrected based on the sequence provided.)	Illumina MiSeq	RDP classifier, UNITE database	[292]
	cheese	E.Z.N.A.^®^ Soil DNA Kit (Omega Bio-tek)	D1 domain (ns-ref)	Illumina MiSeq	internal database for fungi [265]; Basic Local Alignment Search Tool	[313]
	beer	ns-ref	ITS1 region (BITS-B58S3); Titanium^®^ *Taq* DNA polymerase (Takara Bio)	Illumina MiSeq	UNITE database	[152]
	cheese	ZymoBIOMICS DNA Miniprep Kit (Zymo Research)	ITS1 region (BITS-B58S3)	Illumina MiSeq	UNITE database	[314]
2020	tequila (fermentation)	PowerFood™ Microbial DNA Isolation Kit (MoBio)	D1/D2 domain (NL1-LS2); TaKaRa Ex Taq^®^ DNA Polymerase (Takara Bio); PCR-DGGE (separation, extraction reamplification without GC clamp)	scm	NCBI database	[255]
			ITS-5.8S region (ITS1-ITS4)	Illumina MiSeq	Ribosomal Database Project (RPD)	
	laphet	DNeasy PowerFood Microbial Kit (Qiagen)	D1/D2 domain (NL1-LS2); TaKaRa Ex Taq^®^ DNA Polymerase (Takara Bio); PCR-DGGE (separation, extraction reamplification without GC clamp)	scm	ns	[261]
			ITS1 region (ITS1-F-ITS2)	Illumina MiSeq	UNITE database	
	coffee beans	Combination of conventional nucleic acid extraction techniques and DNeasy Blood and Tissue Kit (Qiagen)	ITS1 region (ITS1-F -ITS2) (The forward primer name given in the article has been corrected based on the sequence provided.); AmpliTaq Gold™ 360 DNA Polymerase (Applied Biosystems)	Illumina MiSeq	UNITE database	[294]
	bean paste	ZymoBIOMICS DNA Miniprep Kit (Zymo Research)	ITS2 region (ITS3_KYO2-ITS4)	Illumina HiSeq	UNITE database	[315]
	wine	E.Z.N.A™ Mag-Bind Soil DNA Kit (Omega Bio-tek)	ITS1-ITS2 fragment (ITS1F -ITS2R) (The primer sequences are not given, no reference is provided. The primers used cannot be identified.)	Illumina MiSeq	UNITE database	[316]
	honey	DNeasy PowerSoil DNA Isolation Kit (Qiagen)	ITS2 region (ITS-u3-ITS-u4); Phusion™ High-Fidelity DNA Polymerase (NEB)	Illumina (platform not specified)	UNITE database	[317]

^a^ ns-ref: not specified in the article, but reference is provided; ^b^ ns: not specified in the article.

## Data Availability

No new data were created or analysed in this study.

## Abbreviations

The following abbreviations are used in this manuscript:

NSYs	non-*Saccharomyces* yeasts
CFU	colony forming unit
DT, TTD	detection time, time to detection
ATP	adenosine triphosphate
RLU	relative light units
ELISA	enzyme-linked immunosorbent assay
rRNA	ribosomal ribonucleic acid
ITS	internal transcribed spacer
RT-PCR	reverse transcriptase PCR
PCR	polymerase chain reaction
SIP	surface-imprinted polymer
VBNC	viable but non-culturable
FT-IR	Fourier-transform infrared spectroscopy
GC	gas chromatography
MALDI-TOF MS	matrix-assisted laser desorption/ionisation time-of-flight mass spectrometry
HCCA	α-cyano-4-hydroxycinnamic acid
*m*/*z*	mass-to-charge ratio
rDNA	ribosomal deoxyribonucleic acid
SSU	small subunit
LSU	large subunit
RFLP	restriction fragment length polymorphism
NGS	next-generation sequencing
OTUs	operational taxonomic units
ASVs	amplicon sequence variants
rep-PCR	repetitive sequence-based PCR
RAPD-PCR	randomly amplified polymorphic DNA PCR
BLAST	basic local alignment search tool
qPCR	quantitative real-time PCR
mtDNA	mitochondrial deoxyribonucleic acid
DGGE	denaturing gradient gel electrophoresis
RS	Raman spectroscopy
SERS	surface-enhanced Raman spectroscopy
SCRS	single-cell Raman spectroscopy
DL	deep learning
ANNs	artificial neural networks
DNNs	deep neural networks
CNNs	convolutional neural networks
